# The histone deacetylase *BpHST1* influences branch angle in white birch (*Betula platyphylla*)

**DOI:** 10.1111/tpj.71116

**Published:** 2026-09-13

**Authors:** Lili Hou, Baoxin Li, Yueying Liu, Qingzhu Zhang, Zhimin Zheng

**Affiliations:** ^1^ State Key Laboratory of Tree Genetics and Breeding, College of Forestry Northeast Forestry University Harbin 150040 China; ^2^ The Center for Basic Forestry Research, College of Forestry Northeast Forestry University Harbin 150040 China; ^3^ School of Ecology Northeast Forestry University Harbin China

**Keywords:** birch architecture, *BpHST1*, histone acetylation, H3K27

## Abstract

Optimization of tree architecture is essential for advancing sustainable forestry, as appropriate canopy structure can contribute to growth and development. However, the molecular mechanisms that control tree architecture have not been fully characterized. While the putative NAD‐dependent histone deacetylase HST1 was shown to regulate branching architecture in multiple crop species, whether epigenetic mechanism also influence tree architecture is unknown. Here, we investigated the genetic regulation of branching angle in white birch (*Betula platyphylla* Suk.), a pioneer tree of the temperate and boreal forests. Using *bphst1* mutants generated via gene editing, we demonstrate that BpHST1 is involved in tree architecture by modulating branching angles. We provide evidence that BpHST1 is a histone deacetylase associated with altered H3K27ac levels and that it affects the expression of the cellulase gene *CELLULASE 1* (*BpCEL1*). Compared with the wild type*, BpHST1* knockout and *BpCEL1* overexpression both resulted in reduced branch angles, supporting *BpCEL1* as a candidate downstream effector of BpHST1. Our study suggests that BpHST1 influences the birch architecture by altering H3K27 acetylation at the *BpCEL1* locus, elucidating a previously unknown aspect of the epigenetic mechanisms that control tree architecture in white birch.

## Significance Statement

The histone deacetylase BpHST1 is associated with H3K27ac modulation at the cell‐wall biosynthesis gene *BpCEL1* and modulates branching angle. This work reveals a novel epigenetic pathway governing birch branch angle, connecting histone deacetylation with woody plant branch development and providing molecular targets for forest architecture optimization.

## INTRODUCTION

Plant architecture, defined by the integrated manifestation of traits, such as plant height, branch number, and branch angle, directly governs productivity (Hollender & Dardick, 2014; Su et al., 2023). The optimization of canopy architecture is therefore a promising strategy for enhancing yield (Xia et al., 2021). Branch angle, a key architectural trait, determines the spatial arrangement of the canopy, the efficiency of light energy capture, and adaptability to high planting densities. Together, these characteristics influence photosynthetic performance and final yield through mechanisms that are widely conserved in species, such as rice (*Oryza sativa*) (Wang et al., 2022), maize (*Zea mays*) (Tian et al., 2024), tomato (*Solanum lycopersicum*) (Dong et al., 2023), and peach (*Prunus persica*) (Zhang et al., 2025).

Tree architecture is governed by a complex regulatory network in which key components, such as *TAC1* (*TILLER* *ANGLE CONTROL* *1*), *LAZY1*, and *WEEP*, translate genetic programs and environmental signals into morphological outcomes. Hill and Hollender discussed the genetic basis of variation in plant architecture from the standpoint of plant height and crown width and proposed a hypothetical model for the regulation of branch angle (Hill & Hollender, 2019). However, owing to differences in gene copy number variation among these regulatory genes, the regulation of architecture may vary among plant species (Gioppato & Dornelas, 2021). The detailed molecular mechanisms that enable architecture optimization have yet to be characterized and translated into practical applications, particularly in forest trees. A compact and upright canopy architecture is compatible with high‐density planting while promoting high photosynthetic rates and nutrient use efficiency, providing a foundation for improving tree architecture and wood yield (Choi et al., 2025; Xu et al., 2017; Zheng et al., 2022). The narrow‐crown phenotype is a valuable breeding target that has been shown to improve wood yield and quality in *Populus* (Xu et al., 2017), although its molecular basis remains to be clarified.

**Figure 1:**
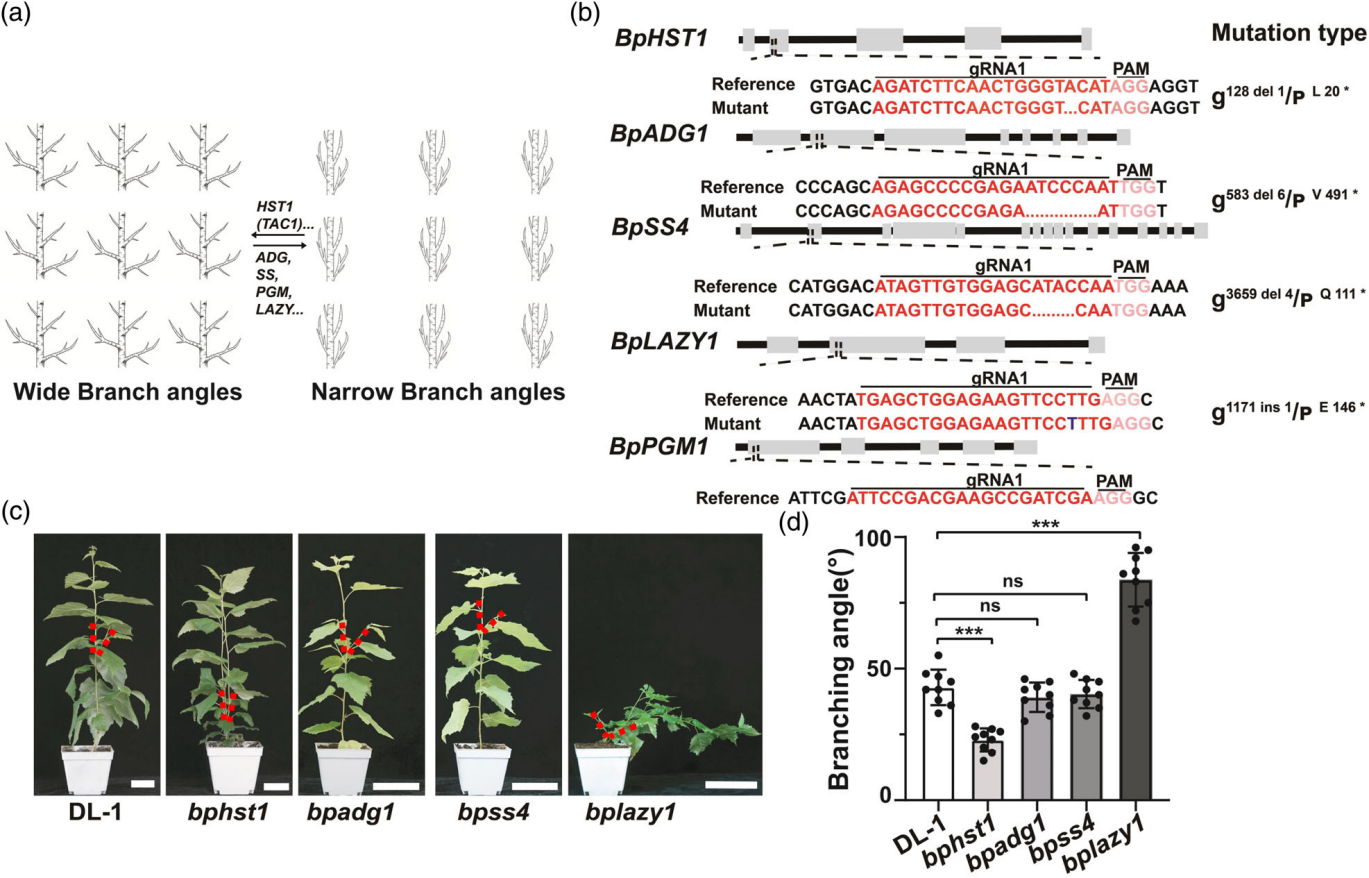
Construction of an architecture‐related mutants in birch using CRISPR/Cas9. (a) Examples of genetic regulation of tree architecture. (b) Mutation site analysis of architecture‐related genes. Red letters represent the sgRNA reference sequence; pale red letters indicate the protospacer adjacent motif (PAM) sequence; ellipsis with red denote deletions; del. for deletion; The ‘g121 del 1’ and ‘p L 20 *’ represent genomic 121st deletion 1 bp, and protein 20th Leucine substituted to termination codon, respectively; V: Valine, Q: Glutamine, E: Glutamic acid. (c) Phenotypic comparison of DL‐1 and tree architecture‐related genes. The red line represents the angle between the main stem and the lateral branch. (d) Phenotypic statistics of mutants in Figure 1c. Data are presented as the mean ± SD, *n* = 9, (***P < 0.001, ns: not significant, *t*‐test).

Several genes, including *LAZY1*, *Homologs of Sirtuins Two 1* (*HST1*), *ADG* (*ADP‐glucose pyrophosphorylase*), and *SS* (*starch synthase*), have been shown to integrate gravity, light, and auxin signaling pathways to control shoot and crown architecture, making them promising targets for trait improvement (Hill & Hollender, 2019; Xie et al., 1999; Yoshida et al., 1988) (Figure 1a). The roles of these genes are well established in crops and fruit trees, but whether their functions are conserved among forest trees remains to be determined (Dardick et al., 2013; Yin et al., 2025; Yu et al., 2007; Zhao et al., 2022). In particular, TAC1 contributes to the regulation of narrow architecture (Wang, Tu, et al., 2023). First identified in rice, where it conferred a compact architecture with extremely erect tillers (Yu et al., 2007), TAC1 plays a conserved role in regulating plant architecture in rice (Yin et al., 2025), Arabidopsis (*Arabidopsis thaliana*) (Waite & Dardick, 2018), hybrid poplar (Choi et al., 2025), soybean (*Glycine max*) (Cai et al., 2026), cotton (*Gossypium hirsutum*) (Kangben et al., 2024), and other species. Structural predictions indicate that TAC1 may contain an HST1‐like NAD‐dependent deacetylase domain (Zhao et al., 2022), leading to its designation as HST1 in wheat, but its molecular mechanism remains unclear.

Epigenetic modifications, including DNA methylation, histone modifications, and small RNAs, modulate the expression of key developmental genes in response to environmental stimuli, enabling adaptive growth (Tang et al., 2017; Yang et al., 2020; Zhang et al., 2019). For example, *plant architecture 1* (*par1*) in maize is an epiallele resulting from methylation‐dependent regulation of an insertion of a CACTA transposon in *Par1*: lower methylation levels at this CACTA insertion induce dwarfism, narrow leaves, and erect leaf angles (Li et al., 2025). In cucumber (*Cucumis sativus*), the N‐terminal domain of the TCP‐type transcription factor TENDRIL‐LESS (TEN) exhibits intrinsic histone acetyltransferase activity that activates transcription, thereby modulating shoot architecture (Yang et al., 2020).

White birch (*Betula platyphylla* Suk.) is found predominantly in temperate and boreal forests of the Northern Hemisphere and has significant economic and ecological value (Cheng et al., 2023; Tan et al., 2020). Here, we used clustered regularly interspaced short palindromic repeats (CRISPR)/CRISPR‐associated nuclease 9 (Cas9)‐mediated gene editing to generate mutants of several key genes known to regulate plant architecture in other species. *bphst1* mutants had significantly narrower branch angles than the wild‐type DL‐1, whereas *bplazy1* mutants had wider branch angles and a prostrate growth habit. We extended our investigation to the histone deacetylase HST1 and how it regulates tree architecture, providing evidence for its role in the epigenetic regulation of gene expression. Our findings reveal that BpHST1 affects the expression of *CELLULASE 1* (*BpCEL1*) through its modulation of H3K27 acetylation, thereby controlling branch angle. Our findings provide insight into the epigenetic regulation of tree architecture and suggest molecular breeding strategies for forest trees.

## RESULTS

### CRISPR/Cas9 mutants of architecture‐related genes in birch

We used CRISPR/Cas9 gene editing to introduce mutations in birch homologs of genes known to control plant architecture in other species: *HST1*, *ADG1*, *SS4*, *LAZY1*, and *PGM1*. We obtained mutants for all genes except *BpPGM1* (Figure 1b). Phenotypic characterization revealed that the *bphst1* mutant has significantly narrower branch angles than the wild‐type DL‐1, whereas the *bplazy1* mutant had wider branch angles and a prostrate growth habit (*P* < 0.001, Student's *t*‐test); the *bpadg1* and *bpss4* mutants were similar in architecture to the DL‐1 (Figure 1c,d). Because rapid growth and superior wood quality are important breeding objectives, we focused on the molecular mechanism by which mutation of *BpHST1* confers a narrow‐branching phenotype. Since its initial identification in rice (Yu et al., 2007), *HST1* has been shown to play a conserved role in plant architecture across multiple species; however, its precise molecular mechanism remains unclear (Table 1).

**Table 1:** *TAC1* regulates the branch angle in different species

Code	Gene names	Species	Description	References
1	*OsTAC1*	*Oryza sativa*	Control tiller angles	Yu et al. (2007)
				Yin et al. (2025)
2	*PpTAC1*	*Prunus persica*	Influence axillary shoot growth angles	Dardick et al. (2013)
3	*AtTAC1*	*Arabidopsis thaliana*	Modulate plant architecture	Waite and Dardick (2018)
4	*TaHST1L*	*Triticum aestivum*	Control wheat tiller angles	Zhao et al. (2022)
	*(TaTAC1)*			
5	*GmCSA1*	*Glycine max*	Enhance shoot compactness	Cai et al. (2026)
	*(GmTAC1)*			
6	*PtTAC1*	*Populus trichocarpa*	Regulate upright branch angles	Choi et al. (2025)

### BpHST1 is involved in regulating branch angle

**Figure 2:**
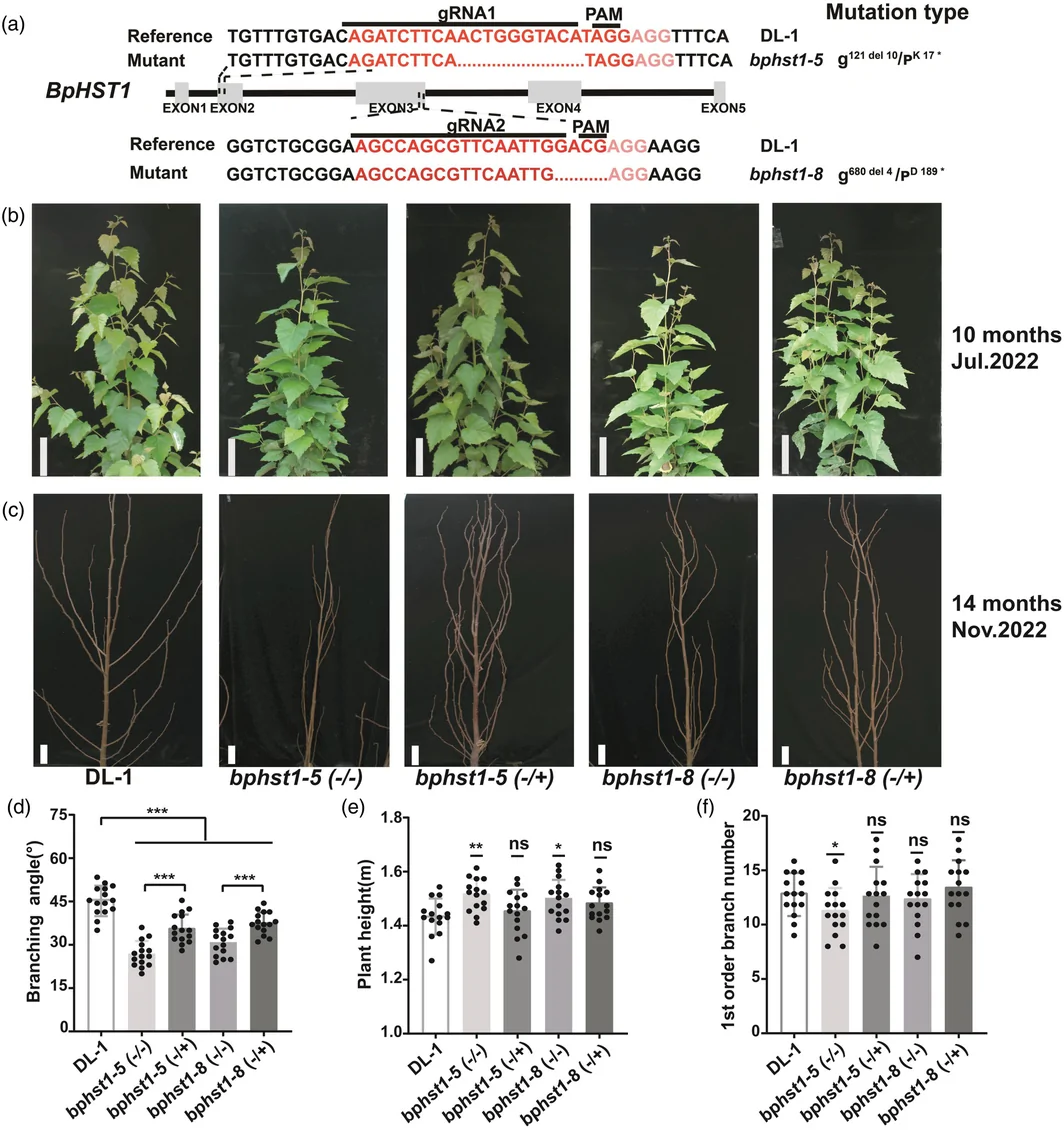
BpHST1 is involved in regulating branch angle. (a) Mutation site analysis of *bphst1*. Red letters represent the sgRNA reference sequence; pale red letters AGG indicate the PAM (Protospacer Adjacent Motif) sequence; ellipsis with red denote deletions; del. for deletion; The ‘g121 del 10’ and ‘p K 17 *’ represent genomic 121st deletion 10 bp, and protein 17th Lysine substituted to termination codon, respectively; K: Lysine; D: Aspartic acid. (b, c) Phenotypic comparison of DL‐1 and four *bphst1* plants (*bphst1‐5*(−/−), *bphst1‐5*(−/+), *bphst1‐8*(−/−), *bphst1‐8*(−/+), Bar = 10 cm. (d–f) Phenotypic statistics of 14‐month‐old DL‐1 and *bphst1* plants. Data are presented as the mean ± SD, *n* = 15, (*P < 0.05; **P < 0.01, ***P < 0.001, ns: not significant, *t*‐test).

**Figure 3:**
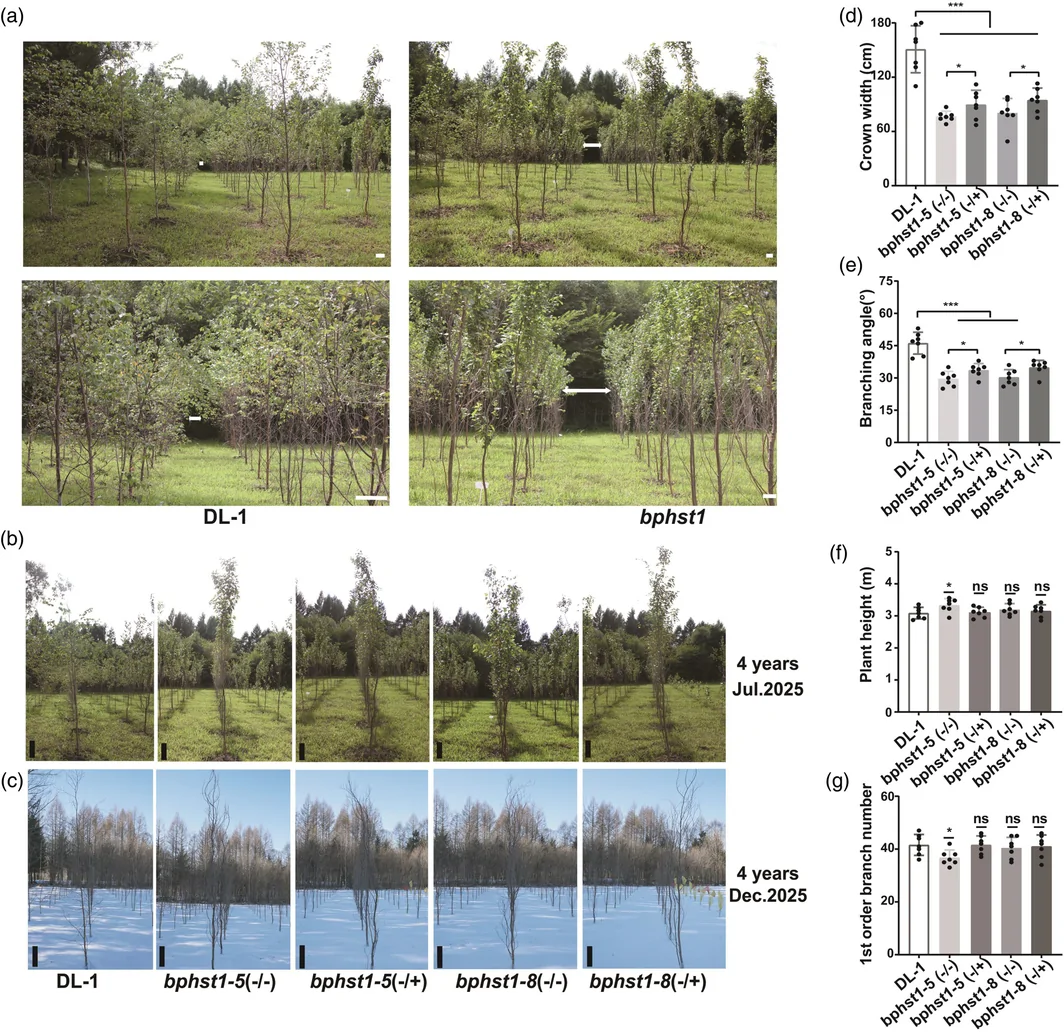
Phenotypes of DL‐1 and *bphst1* in the field. (a) Phenotype of 4‐year‐old DL‐1 and *bphst1* plants (Long shot and close up), the white line represents the distance between two crown widths, plant spacing is 2 m*2 m, scale bar = 0.1 m. (b, c) Phenotype of 4‐year‐old DL‐1 and *bphst1* plants. Scale bar = 0.3 m. (d–g) Phenotypic comparison of DL‐1 and *bphst1* of the shown in b, Data are presented as the mean ± SD, *n* = 7, (*P < 0.05, ***P < 0.001, ns: not significant, *t*‐test).

We selected a new CRISPR/Cas9 target site in the third exon to generate weaker *bphst1* mutant alleles for phenotypic and functional analysis alongside the strong mutant *bphst1‐5* (Figure 2a). We obtained 64 independent plants harboring single homozygous mutations in *BpHST1* (Figure S1) and transferred all plants to Mao'ershan Experimental Forest Station (45°20′ N, 127°30′ E) for field observations. Based on sequencing information, we selected homozygous or heterozygous plants carrying the strong allele *bphst1‐5*(*−/−*) and *bphst1‐5*(*−/+*) or the weaker allele *bphst1‐8*(*−/−*) and *bphst1‐8*(*−/+*) for phenotypic observation. At the 1‐year stage, all *bphst1* mutants displayed smaller branch angles (*P* < 0.001, *t*‐test) than DL‐1 (Figure 2b–d). The homozygous mutants were also higher (*P* < 0.01, *t*‐test) and, in the case of *bphst1‐5*(*−/−*), produced fewer first‐order branches (*P* < 0.05, Student's *t*‐test) (Figure 2e,f). The intermediate phenotypes of heterozygous plants indicated a dosage‐dependent control of the branching trait (*P* < 0.001, Figure 2d). Phenotypic analysis of 4‐year‐old trees revealed that both homozygous and heterozygous plants had significantly smaller crown widths and narrower branching angles than DL‐1 (*P* < 0.001, Figure 3a–e), whereas only *bphst1‐5* plants were significantly higher and had fewer first‐order branches (Figure 3f,g). These results demonstrate that *BpHST1* influences birch architecture through its effect on branching angle.

### BpHST1 exhibits deacetylase activity and is associated with altered H3K27ac levels

**Figure 4:**
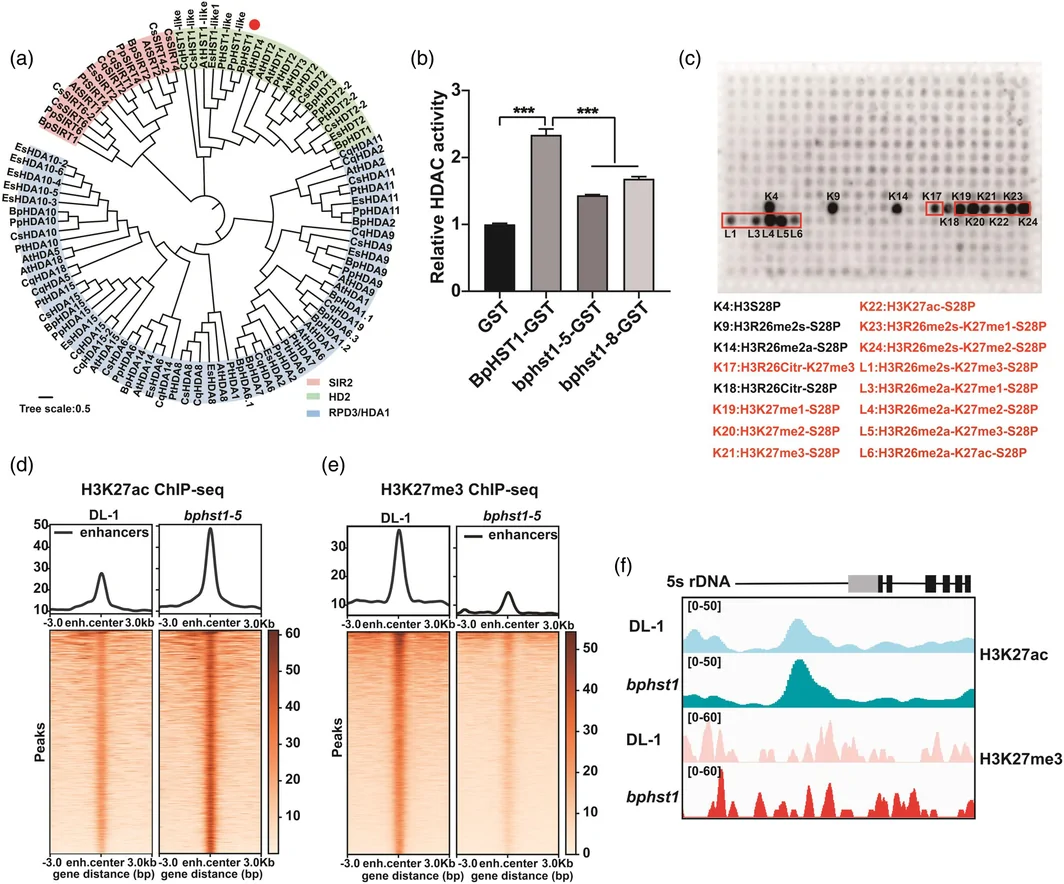
BpHST1 exhibits histone deacetylase activity and is associated with altered H3K27ac levels. (a) Phylogenetic tree of *HST1* genes in white birch, Arabidopsis, poplar, quinoa, tea, rice, peach and eucalyptus. HDACs in all eukaryotes are grouped into three families: RPD3/HDA1 (Reduced Potassium Dependence 3/Histone Deacetylase 1), SIR2 (Silent Information Regulator 2), and HD2 (Histone Deacetylase 2)‐related protein families. (b) HDAC activity assay. Enzymatic activity was shown as relative. The data are shown as mean ± SD, *n* = 3. Asterisks represent significant differences (*P < 0.05; Student's *t*‐test). (c) Analysis of histone modification peptide array using GST monoclonal antibody. GST: glutathione‐S‐transferase. The meaning of the red box and the red mark is to indicate the related modifications that are associated with the H3K27 site. (d) Heatmap showing the corresponding histone deacetylation levels of the H3K27ac peaks of DL‐1 and *bphst1‐5*. (e) Heatmap showing the corresponding histone methylation levels of the H3K27me3 peaks of DL‐1 and *bphst1‐5*. (f) ChIP‐seq of H3K27ac, H3K27me3 on 5 s rDNA. Gray boxes, black boxes and black lines represent the 5′‐UTR, exons and introns, respectively. Transcription start sites (TSS) are presented with arrows.

Previous studies have suggested that rice HST1 may be an NAD‐dependent deacetylase (Wang, Gu, et al., 2023; Zhao et al., 2022). Our phylogenetic analysis placed BpHST1 in a clade with HST1‐like proteins from multiple plant species (Chen et al., 2025), indicating that it is a member of the larger histone deacetylase family (Figure 4a). Subcellular localization analysis demonstrated that BpHST1‐GFP localizes mainly to the nucleus, consistent with a role in histone deacetylation (Figure S2). HDAC activity assays with recombinant purified BpHST1 fused to glutathione‐S‐transferase (GST) showed that the deacetylase activity of full‐length HST1 is much higher than that of the GST control (*P* < 0.001, Figure 4b); similarly, BpHST1 truncation mutants consisting of 17 or 189 amino acids had significantly lower HDAC activity than the full‐length protein. These results provide strong evidence for the HDAC activity of BpHST1. We tested the interaction of recombinant purified BpHST1‐GST with various histone peptide modifications using a MODified Histone Peptide Array (Table S1). After incubation of the array with BpHST1‐GST, followed by an anti‐GST monoclonal antibody for detection of the GST‐tagged protein, we obtained 16 high‐intensity signals (Figure 4c). Based on quantified signal intensities, we identified the eight histone peptide sites with the highest binding signals (Figure 4c; Figure S3), which included H3S28P, H3R26, and H3K27. Therefore, we focused on the role of H3K27 modification in birch branching development.

Many studies have revealed key regulatory roles of H3K27 in plant development (Lu et al., 2011; Wang, Gu, et al., 2023; Zeng et al., 2025), and a recent report suggested that SIRT7 (the homolog of HST1) may potentially regulate the H3K27me3 modification (Simonet et al., 2026). To investigate how BpHST1 regulates H3K27 modification, we selected young leaves of 2‐month‐old DL‐1 and *bphst1‐5*(*−/−*) for chromatin immunoprecipitation followed by sequencing (ChIP‐seq) with an anti‐H3K27ac antibody. We identified 18 437 significant H3K27ac peaks in DL‐1 and 17 180 in *bphst1‐5* (Figure S4A,B). Based on a scatterplot comparing H3K27ac peak levels in the two genotypes, we identified 1720 BpHST1‐specific peaks, 15 460 shared peaks, and 2977 DL‐1‐specific peaks. Overall, ChIP‐seq analysis revealed higher H3K27ac levels in *bphst1‐5* plants than in DL‐1 (*P* < 0.05, Figure 4d), demonstrating that BpHST1 decreases H3K27ac levels. H3K27 acetylation, which is associated with transcriptional activation, is mutually exclusive with H3K27me3 methylation, which is associated with transcriptional repression (Maleszewska et al., 2018). Given the relationship between these two histone marks, we also performed ChIP‐seq for H3K27me3 on young leaves of DL‐1 and *bphst1‐5* plants (Figure S4C). We identified 9494 significant H3K27me3 peaks in DL‐1 and 4209 in *bphst1‐5*: 1486 *bphst1*‐specific peaks, 2723 shared peaks, and 6771 DL‐1‐specific peaks. ChIP‐seq analysis showed that H3K27me3 levels are significantly lower in *bphst1‐5* than in DL‐1 (*P* < 0.01, Figure 4e). Thus, H3K27ac and H3K27me3 levels appear to be inversely associated, with higher acetylation and lower methylation in genomic regions of the *bphst1* mutant (Figure S5), indicating that BpHST1 function is associated with altered H3K27 modification, especially in repetitive regions, such as 5S ribosomal RNA (rDNA) (Figure 4f). The relationship between BpHST1, H3K27ac, and H3K27me3 remains to be tested.

### *BpCEL1* acts as a candidate downstream effector of BpHST1

**Figure 5:**
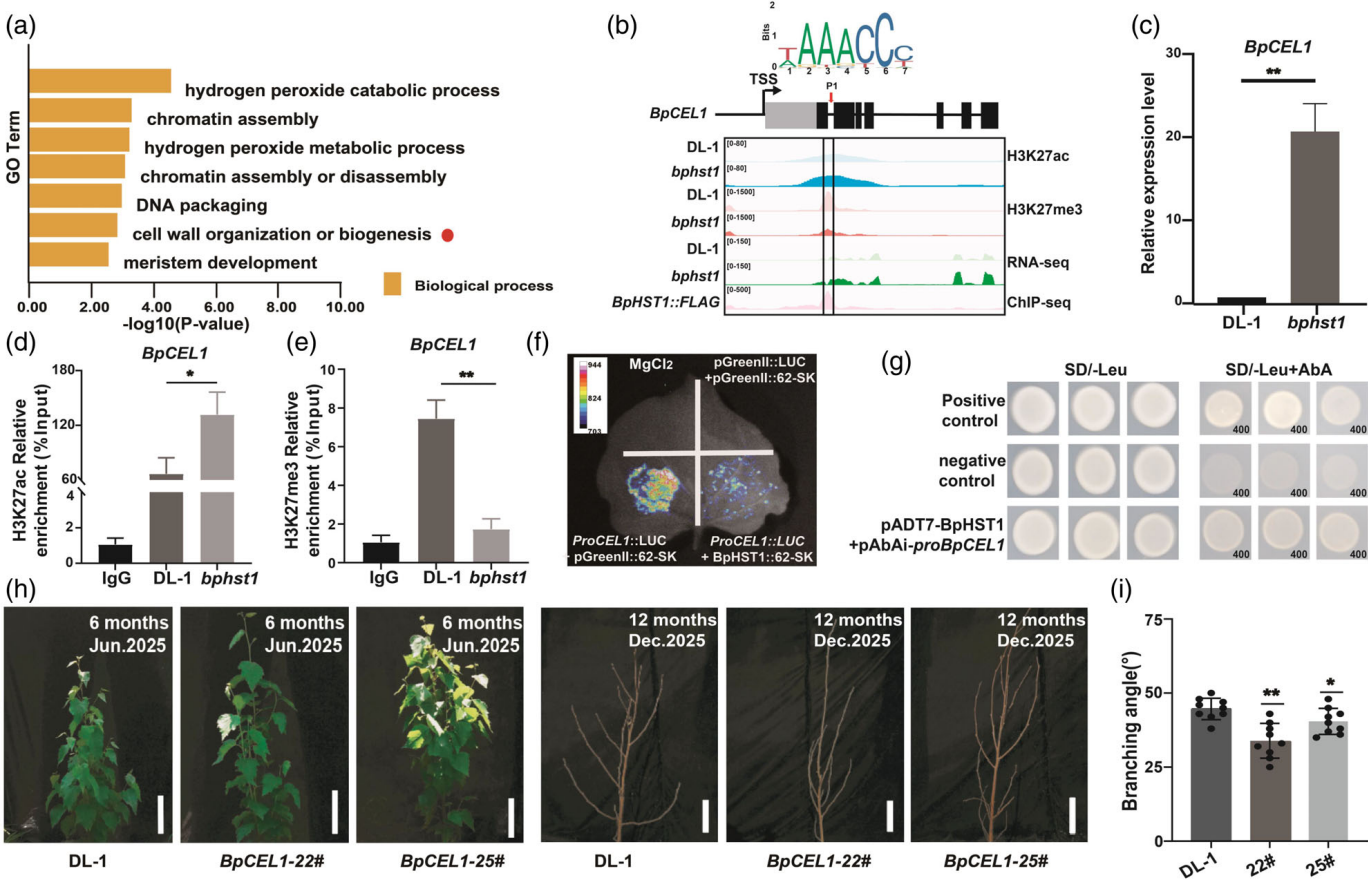
*BpCEL1* acts as a candidate downstream effector of BpHST1. (a) Gene Ontology enrichment analysis for DEGs of biological processes. The red circle represents the candidate term. (b) IGV illustrating the reproducible peaks of H3K27ac, H3K27me3 and *BpHST1*::FLAG ChIP‐seq and RNA‐seq at the *BpCEL1*. The enriched motif were analyzed by MEME. The red arrow represents the position of motif binding site. Gray boxes, black boxes, and black lines represent the 5′‐UTR, exons, and introns, respectively. Transcription start sites (TSS) are presented with arrows. (c) The relative expression levels of *BpCEL1* in DL‐1 and *bphst1*. **P < 0.01; *t*‐test. (d, e) *BpCEL1* enrichment on the tested H3K27ac and H3K27me3 levels in DL‐1 and *bphst1* by ChIP‐qPCR. IgG was served as the negative control. (*P < 0.05, **P < 0.01; *t*‐test). (f) BpHST1 interacts with *BpCEL1* promoter in LUC assay. The pseudocolor bar showed the luminescence intensity in the image. MgCl_2_: 10 mM. (g) Yeast one‐hybrid assay for BpHST1 binding to the regulatory regions of the *BpCEL1* promoter. The number in the downer right corner indicated Aureobasidin A concentration (AbA; ng/mL). (h) Comparative phenotypes of 6‐month‐old and 12‐month‐old DL‐1 and *35S::BpCEL1* overexpression lines (22# and 25#). Bar = 10 cm. (i) The branch angle of the 6‐month‐old genotypes shown in (H). Data are presented as the mean ± SD, *n* = 9. Asterisks represent significant differences (*P < 0.05, **P < 0.01; *t*‐test).

To identify potential regulatory targets of BpHST1, we compared the transcriptome profiles of DL‐1 and *bphst1‐5* (*−/−*) *and bphst1‐8* (*−/−*) through transcriptome sequencing (RNA‐seq) (Figure S6). As previous studies have reported that loss of deacetylase function leads to transcriptional activation (An et al., 2024; Le et al., 2025), we focused on 269 genes that were significantly upregulated in *bphst1* compared with DL‐1 (Figure S6). Gene Ontology (GO) analysis of these 269 genes identified significantly enriched terms, such as hydrogen peroxide catabolic/metabolic processes, chromatin assembly/disassembly, DNA packaging, and cell‐wall organization/biogenesis (Figure 5a). Previous studies have shown that genes related to cell walls in Arabidopsis and rice can affect the angle of branching (tiller) (Notaguchi et al., 2020; Ohmiya et al., 2003), we focused on genes involved in cell‐wall organization or biogenesis (GO:0071554). Because BpHST1 is a deacetylase, we performed a *de novo* motif analysis using the H3K27ac ChIP‐seq data of BpHST1‐specific peaks. H3K27ac peaks were enriched in gene regions, which accounted for approximately 63.1% of all peaks (coding regions and introns); smaller proportions were located in transposable element regions (27%), promoter regions (6.9%), and intergenic regions (3%) (Figure S7A). *De novo* motif analysis revealed a significantly enriched motif (Figure 5b) that was specifically enriched in gene regions compared with other genomic contexts (Figure S7B). An integration of the ChIP‐seq and RNA‐seq data revealed that this predicted motif is present in five genes annotated as being related to the GO term ‘cell‐wall organization or biogenesis’, including the cellulase gene *BpCEL1*. *BpCEL1* also exhibited increased acetylation levels and lower methylation levels in the *bphst1‐5(−/−)* mutant (Figure 5b; Figure S8). Reverse transcription quantitative PCR (RT‐qPCR) of 12 genes related to cell‐wall organization or biogenesis (GO:0071554) revealed that eight (*XTH16*, *FLA11*, *TBL19*, *CEL1*, *XTH32*, *TBL36*, *CSLG3*, and *FLA11‐2*) are significantly upregulated in the *bphst1‐5* (*−/−*) mutant, and *BpCEL1* exhibited the greatest increase in expression (20‐fold, *P* < 0.01; Figure 5c; Figure S9). ChIP‐seq with an anti‐FLAG antibody on *35S:BpHST1‐FLAG* seedlings revealed enrichment of BpHST1 in the *BpCEL1*, where the enriched H3K27ac motif was located (Figure 5b; Figures S8 and S10). Based on these findings, we postulate that *BpCEL1* acts as a candidate downstream effector of BpHST1, mediating branching angle.

To investigate whether *BpCEL1* expression is under epigenetic regulation, we performed ChIP‐qPCR to assess the enrichment of H3K27ac and H3K27me3 at the *BpCEL1* locus in DL‐1 and *bphst1‐5* (−/−). Compared with DL‐1, the *bphst1* mutant had a significantly higher H3K27ac level and a significantly lower H3K27me3 level at the *BpCEL1* locus (*P* < 0.05, Figure 5d,e). Consistent with the ChIP‐qPCR analysis, dual‐luciferase reporter assays and yeast one‐hybrid experiments collectively support the identification of *BpCEL1* as a downstream candidate effector of BpHST1‐mediated epigenetic regulation (Figure 5f,g; Figure S11). The potential involvement of additional recruited factors in this regulatory process remains to be further investigated.

### *BpCEL1* overexpression results in smaller branching angle

We examined the effect of *BpCEL1* on branching angle by introducing the full‐length coding sequence of *BpCEL1* driven by the *35S* promoter into DL‐1. We obtained five independent transgenic plants with significantly higher *BpCEL1* expression levels than DL‐1 (*P* < 0.05; Figure S12) and much narrower branching angles than DL‐1 at 6 months of age (*P* < 0.05, *P* < 0.01, Figure 5h,i). We selected the two transgenic plants with the highest and lowest expression levels of *BpCEL1* (*BpCEL1*‐OE22# and *BpCEL1*‐OE25#) and analyzed their cell‐wall composition, focusing on the quantification of three major structural polymers. Compared with DL‐1, *BpCEL1*‐OE22# accumulated significantly higher hemicellulose and lignin levels and significantly lower cellulose content (*P* < 0.05, Figure S13), whereas *BpCEL1*‐OE25# had significantly higher lignin content only. Interestingly, we found that the changes in cell‐wall components of the *bphst1* mutants were consistent with the *BpCEL1*‐OE22# (Figure S14). Analysis of longitudinal lateral branch sections revealed that the β angle (defined as the angle between the lateral branch and the main stem) is significantly smaller in *BpCEL1*‐OE22# and *bphst1‐5*(−/−) than in DL‐1 (*P* < 0.01, Figure S15A–C). The cells in the stems of *BpCEL1*‐OE22# and *bphst1* plants were also longer and wider than DL‐1 (*P* < 0.05, Figure S15D,E). These cellular changes indicate that the branch angles in both the *BpCEL1* overexpression line and the *bphst1* mutant are reduced, suggesting that *BpCEL1* acts as a candidate effector factor that can influence the branch angle. However, the causal relationship between the changes in cell‐wall components/cell morphology and the changes in branch angle requires further investigation.

## DISCUSSION

Although acetylation and methylation predominantly target lysine residues in histones, acetylation typically occupies these sites first due to the inherent greater binding affinity of acetyl groups to lysine (Jacquet et al., 2016). Consequently, deacetylation is thought to serve as a prerequisite step, while transcriptional repression is ultimately mediated by methylation of H3K27 (Xiao et al., 2003). Our results showed that the *bphst1* mutants displayed increased H3K27ac and decreased H3K27me3 (Figure 4d,e). H3K27 acetylation and methylation are well known to function antagonistically. Mechanistically, HAT‐catalyzed H3K27 acetylation (e.g., by CBP/p300) causes steric hindrance, which blocks the PRC2/EZH2 complex from methylating the same histone residue (Bromberg et al., 2021; Pasini et al., 2010). Furthermore, accumulated H3K27ac recruits KDM6A/B demethylases to erase pre‐existing H3K27me3, further lowering local H3K27me3 abundance (Ding et al., 2025; Guccione et al., 2007). Although the relationship between these two modifications has been reported in other studies, the current data do not demonstrate that BpHST1 is involved in the deposition of H3K27me3 or the recruitment of PRC2 in birch. Future studies exploring how BpHST1 regulates this cascade and verifying its interaction with the PRC2 complex will help clarify the dynamic balance of H3K27 epigenetic modifications in birch.

Histone deacetylases (HDACs) exert epigenetic regulation via deacetylating both histones and non‐histone proteins, thereby modulating transcription, translation and cellular metabolism. Distinct epigenetic marks act synergistically to remodel chromatin and control the expression of downstream genes (Baroux et al., 2011; Miryeganeh, 2022). Transcriptional regulators mediate the targeted recruitment of histone deacetylases (HDACs) to specific genomic sites, leading to the assembly of localized repression complexes (Kiermaier & Eilers, 1997). For instance, MYB‐like transcription factors assemble a repressive complex with the corepressor TOPLESS and HDA19, leading to lower H3K9ac levels at the *MIR159* promoter and downregulated expression (Jing et al., 2023). Similarly, the transcription factor TCP15 recruits the histone deacetylase HDA4 to form a functional complex that represses downstream gene expression by lowering H3K9ac levels, thereby inhibiting axillary bud outgrowth and ultimately promoting a more compact plant architecture in tomato (Song et al., 2025). In addition to classical transcription factor‐driven control, the epigenetic state of several genes control plant architecture, especially through histone modifications (Li et al., 2025). In tomato, the histone deacetylase HDA4 binds directly to the promoters of *PHYTOCHROME B1* (*PHYB1*) and *INDOLE‐3‐ACETIC ACID INDUCIBLE 12* (*IAA12*), lowering their H3K9 acetylation levels to repress their transcription, thereby inhibiting lateral bud outgrowth and promoting a compact plant architecture (Song et al., 2025). Beyond transcription factors, additional chromatin‐associated proteins may recruit HST1, including chromatin remodelers (e.g., SWI/SNF) and epigenetic modifier enzymes (e.g., methyltransferases) (Davalos & Esteller, 2010; Mardinian et al., 2021). These findings demonstrate coordinated interplay between distinct epigenetic modifications that collectively modulate chromatin states to regulate architecture.

**Figure 6:**
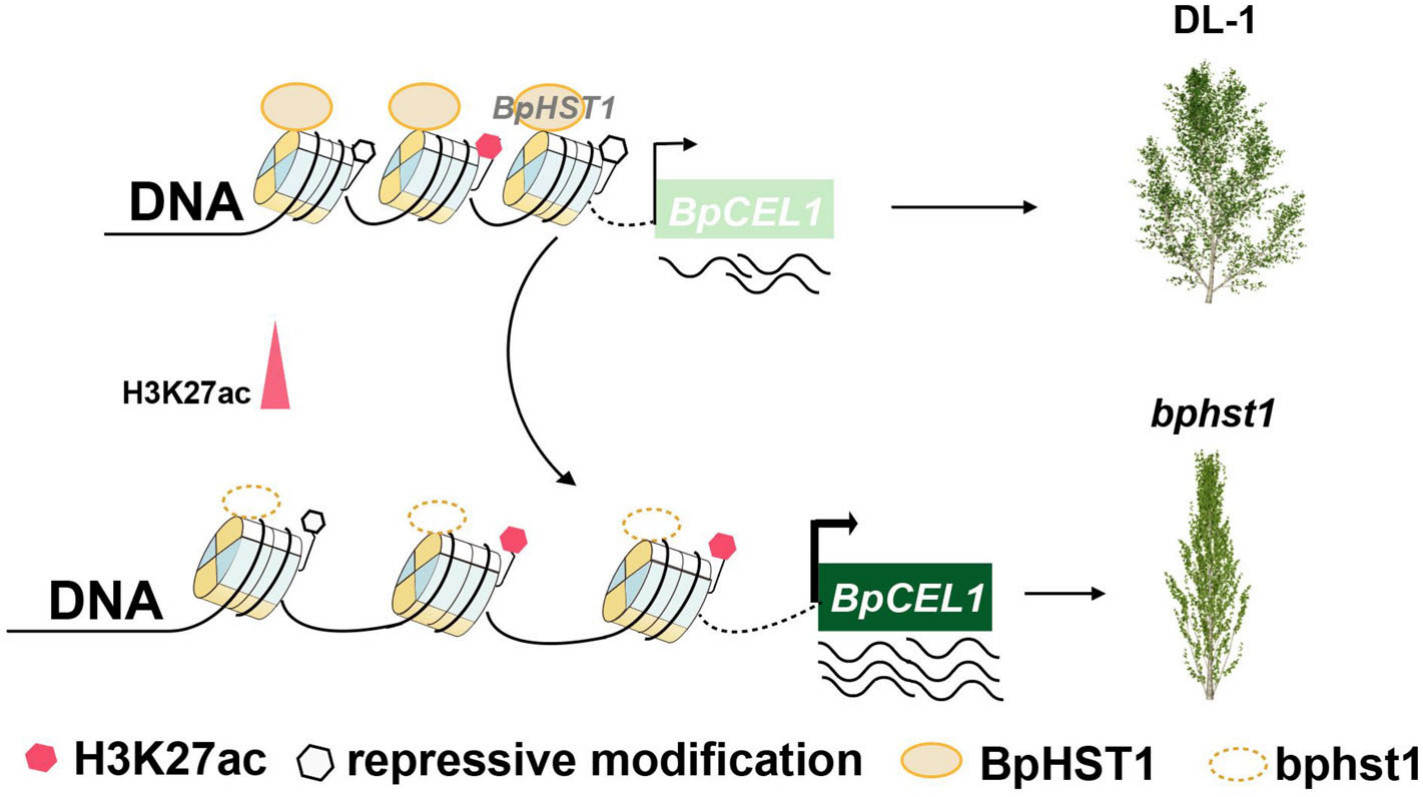
Model of BpHST1‐mediated branch angle regulation via *BpCEL1*. BpHST1 is associated with altered H3K27ac level at the *BpCEL1*, which epigenetically affects the expression of *BpCEL1*, consequently influences the branching angle in birch. Dashed arrows with question marks indicate inferred relationships requiring further validation. The thickness of the arrows is proportional to the relative transcription level.

Although studies have shown that HST1 is an NAD‐dependent deacetylase, its role in epigenetic regulation is not fully understood (Wang, Tu, et al., 2023; Zhao et al., 2022). Here, we provide evidence that BpHST1 is an NAD‐dependent deacetylase (Figure 4a,b) and is associated with H3K27ac modification at the *BpCEL1* locus, correlating with changes in branching angle. *CEL1*, a member of the Glycosyl hydrolase 9B (GH9B) family, contributes to cell‐wall biosynthesis and cellulose metabolism during plant growth (Notaguchi et al., 2020). Previous studies have demonstrated that *CEL1* is essential for proper cell‐wall assembly and cell elongation and plays crucial roles in leaf development and cellulose biosynthesis in poplar trees (Nicol et al., 1998; Ohmiya et al., 2003). The increased lignin and hemicellulose content, along with reduced cellulose in *BpCEL1* overexpression lines, suggests that *CEL1* activity may influence cell‐wall compositional changes and biosynthesis, potentially through altered substrate availability or signaling, consistent with previous observations in poplar (Ohmiya et al., 2003). However, how these cell‐wall changes lead to altered branch angles remains to be tested. Our findings demonstrate that BpHST1 is associated with H3K27ac levels at the *BpCEL1*, which epigenetically affects the expression of *BpCEL1*, consequently promoting the branch angle in birch (Figure 6), establishing a connection between epigenetic regulation and architecture development in trees. Thus, future work aimed at identifying the transcription factors that recruit BpHST1 to *BpCEL1* will be essential to fully understand the recruitment mechanism and this is also the focus of our subsequent research.

Modifying plant architecture has been widely applied in crops and woody plants with demonstrated agricultural value (Dong et al., 2023; Su et al., 2023; Tian et al., 2024). However, this approach presents unique challenges in perennial woody plants due to their prolonged life cycles and slow growth rates (Hollender & Dardick, 2014). Branching angle is a key factor determining wood quality and ecological adaptability of white birch. Mutation of *ADG1* or *SS4* results in wider branch angles in Arabidopsis but not in young birch, suggesting functional divergence in perennial trees that warrants further investigation (Cai et al., 2023; Kiss & Sack, 1990). By contrast, *lazy* mutants in both birch and rice exhibit diminished gravitropism together with wider branch angles, a phenotype suitable for applications in landscape forestry or cultivation of potted ornamental plants (Wang, Tu, et al., 2023). Here, we identified a single putative *HST1* ortholog in the birch genome (Figure 4a). Both homozygous and heterozygous *bphst1* harboring premature stop codons exhibited gradual declines in branch angle, demonstrating a dosage‐dependent effect of BpHST1 on branch architecture (Figures 2b, 3c). This consistent phenotype aligns with that observed in other species (Waite & Dardick, 2018; Yin et al., 2025). The histone deacetylase gene *HST1* may represent a promising target for precision molecular breeding due to its conserved role in modulating chromatin architecture through site‐specific histone deacetylation. Recent studies in crops have demonstrated that *HST1* activates transcriptional repression, thereby optimizing plant architecture (Mazza & Ballare, 2015; Tian et al., 2024). However, our current data do not establish a direct causal link between BpHST1 enzymatic activity and the branching phenotype. Further investigation is required to determine whether the branching phenotype is attributable to the enzymatic activity of BpHST1, its non‐catalytic functions, or indirect effects mediated by other regulatory factors. In summary, HST1 modulates birch plant architecture by altering H3K27ac levels, linking histone epigenetic modification to the improvement of tree architecture.

Epigenetic regulation provides new strategies for targeted improvement of tree architecture, creation of novel germplasms, and breeding of elite varieties. The compact, narrow‐crown architecture of *bphst1* mutants provides a valuable germplasm resource for birch breeding. *HST1* could emerge as a highly promising genetic target for the precise improvement of plant architecture.

## MATERIALS AND METHODS

### Plant material and growth conditions

Birch seedlings for up to 4 months of age were cultivated in a growth chamber under controlled conditions (22°C, 16‐h light/8‐h dark photoperiod). The young leaves of 2‐month‐old birch seedlings were harvested, rapidly frozen in liquid nitrogen, and stored at −80°C for use in RNA‐seq and ChIP‐seq analyses. Three biological replicates were used in this experiment. For long‐term phenotypic evaluation, 1‐year‐old and 4‐year‐old wild‐type (DL‐1) and *bphst1* mutant trees were cultivated under natural conditions at the Mao'ershan Experimental Forest Station (45°20′N, 127°30′E) in Heilongjiang Province, China. The Mao'ershan region has a temperate continental monsoon climate with cold, dry winters (−18.5°C mean January temperature) and warm, humid summers (21.3°C mean July temperature); it receives 600–800 mm annual precipitation and supports mixed broadleaf–coniferous forests dominated by birch, *Pinus*, and *Quercus*.

### Total RNA extraction and RT‐qPCR

Total RNA was extracted from samples with an RNAeasy Kit (Cwbio Jiangsu, China); RNA quality (A260/A280 = 1.9–2.1, 28S/18S ratio = 1.8–2.0) was confirmed using a NanoDrop spectrophotometer (Thermo Fisher). First‐strand cDNA was synthesized using a PrimeScript RT Reagent Kit (Takara, Japan), followed by qPCR analysis. Quantitative PCR (qPCR) was performed using gene‐specific primers (Table S2) and UltraSYBR One Step mix (CWBIO, Jiangsu, China) on a 7500 Fast Real‐time PCR System (Applied Biosystems, QuantStudio 3 Real‐Time PCR Instrument, USA). The birch *Tubulin* gene was used as an internal control, and relative gene expression was calculated by the 2^−ΔΔCt^ method (Livak & Schmittgen, 2001). Three biological and technical replicates were performed per gene.

### Plasmid construction and plant transformation

To obtain the CRISPR/Cas9 mutants, the sgRNA targeting *BpHST1* or other candidate genes was assembled into the pSCI CRISPR/Cas9 vector. Equal volumes of forward and reverse primers at 100 μM were heated to 95°C in a water bath and cooled to room temperature to obtain double‐stranded DNA fragments with SpeI sticky ends. The short DNA fragments were then ligated into the linearized pSCI CRISPR/Cas9 vector using T4 ligase (New England Biolabs); the resulting constructs were confirmed by Sanger sequencing (Sangon Biotech, Shanghai, China). The full‐length *BpHST1* coding sequence was amplified from DL‐1 cDNA and inserted into the pCAMBIA1305.1‐FLAG and pBI121‐GFP vectors. Primers are listed in Table S2. Transformants were obtained by Agrobacterium (EHA105)‐mediated embryonic transformation of mature birch zygotic embryos as previously described (Cheng et al., 2023).

### Measurement of birch phenotypes

Plant height was measured with a measuring pole from the soil surface to the apical shoot. Lateral branch angles were determined using a protractor. For 1‐year‐old birch plants, we measured the angles between the main stem and all primary lateral branches, then calculated the average value to represent the branch angle of an individual plant. A total of 15 DL‐1 and *bphst1* mutants were assayed. For 4‐year‐old mature trees, measurements were conducted above and below breast height (1.3 m). We selected 40 upper branches and 40 lower branches for angle quantification, and the mean of the two groups was defined as the branch angle per plant. Seven individual plants of DL‐1 and *bphst1* mutants were used for this analysis. All statistical analyses were performed using an independent samples *t*‐test. Data are presented as mean ± standard error (Mean ± SE). Graphs were generated using the GraphPad Prism software.

### Microscopy observations

Paraffin longitudinal sections (0.5 cm in size) were prepared from the third internode of lateral branches of 2‐month‐old birch plants. Tissues were fixed in formaldehyde–alcohol–acetic acid (FAA) fixative (Coolaber, Beijing, China) and subjected to a low‐temperature vacuum pump for 30 min at 100 kPa. The FAA was then replaced with fresh solution and the vacuum infiltration was repeated four times. Fixed tissues were then dehydrated through a graded ethanol series (50%, 70%, 80%, 95%, and 100% ethanol, 20 min each) at room temperature, cleared in xylene (two changes, 30 min each), and infiltrated with Paraplast (Sigma‐Aldrich) at 65°C for 5 days. The processed samples from lateral branches were then sliced longitudinally to obtain 10 μm sections. Sections were mounted on glass slides, dewaxed in xylene, rehydrated through a descending ethanol series, and stained with 0.01% (w/v) toluidine blue for 2–3 min. After rinsing with distilled water, the sections were dehydrated, cleared, and mounted with neutral resin. Cell morphology was examined and photographed under a light microscope (Carl Zeiss AG, Oberkochen, Germany) to examine cell morphology in the third internode of lateral branches from 2‐month‐old DL‐1, *BpCEL1‐OE22#*, and *bphst1‐5*(−/−).

### HDAC activity assays

HDAC assays were performed using an HDAC Assay Kit (Shfksc, F50201‐A, Shanghai) following the manufacturer's instructions. In brief, purified recombinant proteins were added to the wells of a microtiter plate coated with anti‐HDAC antibody; absorbance values were measured at 450 nm using a microplate reader. A standard curve was generated using the supplied standards according to the manufacturer's instructions. The HDAC activity of each sample was determined by interpolation from the standard curve, and all measurements were performed in triplicate. The HDAC activity of the sample was determined using a standard curve.

### Modified histone peptide array

The MODified Histone Peptide Array is used to test proteins for binding to distinct post‐translationally modified histone peptides. The array contains 59 single‐site histone modifications on the N‐terminal tails of histones H2A, H2B, H3, and H4, arranged into 384 different modification combinations (Active Motif, Catalog No. 13001, USA), with control spots (locations P20‐P24) provided by the manufacturer for background normalization. Detailed information on the 384 histone modification combinations is provided in Table S1. All experiments were performed according to the manufacturer's protocol. Tris‐buffered saline with Tween 20 (TTBS) buffer (10 mM Tris–HCl, pH 7.4, 0.05% [v/v] Tween 20, and 150 mM NaCl) was used for both the sealing solution and the elution solution. The array was incubated with recombinant BpHST1‐GST protein (10 μg/mL in TTBS with 1% BSA) overnight at 4°C with gentle agitation. The array was then washed three times with TTBS (5 min each) to remove unbound protein and incubated overnight at 4°C with a diluted anti‐GST monoclonal antibody (1:1000 dilution, SAB5300159; Sigma, USA). The next day, the array was rinsed three times with TTBS and incubated with rabbit anti‐mouse IgG (HRP) secondary antibody (1:2500 dilution, ab6728; Abcam, Shanghai) for 1 h. After three final washes with TTBS, signals were detected using enhanced chemiluminescence (ECL) substrate (Meilun, Shanghai). Histone modification sites and their corresponding signal intensities were analyzed using the Array Analyze Software (Active Motif; available at https://www.activemotif.com/catalog/668). For each peptide spot, the mean signal intensity was calculated from duplicate spots located on the left and right sides of each array. Relative signal intensities were obtained by subtracting the background signal derived from empty spots (negative controls) on the same subarray, followed by normalization of the mean signal intensity of each peptide spot to the highest signal intensity within that subarray. A peptide spot was considered a positive hit when its normalized signal intensity exceeded the mean signal intensity of the negative controls. The Array Analyze Software processes spot intensity data from peptide arrays and generates corresponding visualization outputs. For reproducibility assessment, two independent biological replicates were performed using separately prepared recombinant BpHST1‐GST protein and independent arrays. Peptides with uneven background staining, weak diffuse signals, or inconsistent signals across the two replicates were eliminated. Only peptide spots showing consistent positive signals in both replicates were considered valid hits.

### Chromatin immunoprecipitation followed by sequencing (ChIP‐seq)

The young leaves of 2‐month‐old DL‐1, *bphst1*, and *35S:BpHST1‐FLAG* seedlings were used for ChIP. The ChIP assay was performed as described previously with slight modifications (Tang et al., 2022). Immunoprecipitation was performed with anti‐H3K27ac (ab4729; Abcam, Shanghai), anti‐H3K27me3 (ab6002; Abcam, Shanghai), and anti‐FLAG antibodies (10 mg per ChIP reaction) (F3040; Sigma, USA). After antibody incubation, immune complexes were collected. After washing, elution, and reverse crosslinking, DNA was recovered by DNA purification. The recovered DNA was sent to Beijing Novogene Company for library construction, sequencing, and ChIP‐qPCR analysis. Low‐quality reads and reads with adapter sequences were removed using fastp (v0.23.2) (Chen et al., 2018), and the clean reads were mapped to the birch reference genome using Bowtie 2 (v2.5.3) (Langmead & Salzberg, 2012). Duplicate reads were removed using Picard (v3.2.0). Peak calling was performed with MACS2 (v2.2.9.1) (Feng et al., 2012), and heatmaps were created using deepTools (v3.5.4) (Ramírez et al., 2016).

### RNA‐seq

The young leaves of 2‐month‐old DL‐1 and *bphst1* seedlings were used for RNA‐seq. Three separate biological replicates were analyzed for each genotype, for a total of nine samples. Libraries were constructed, subjected to quality controls, and sequenced on an Illumina NovaSeq 6000 system by Biozeron, Shanghai. The HISAT2 plugin of TBtools (Chen et al., 2023) was used to remove adapters, reads with Ns, and low‐quality reads, and to map the clean reads to the white birch reference genome. Gene expression levels were normalized as fragments per kilobase of transcript per million mapped reads (FPKM) values. Differentially expressed genes between WT DL‐1 and the *bphst1* mutant were identified using a false discovery rate of 0.05 and a |log_2_(fold‐change)| value of ≥1.

### Dual‐luciferase assays

The full‐length coding sequence of *BpHST1* was isolated from cDNA prepared from total RNA extracted from birch leaves and cloned into the pGreenII62‐SK effector vector. The promoter and full‐length coding sequence of *BpCEL1* were cloned into the pGreenII 0800‐LUC reporter vector. The resulting constructs were individually introduced into Agrobacterium strain GV3101 (pSoup‐p19) (WeDi, Shanghai, China). Cultures from positive Agrobacterium colonies were resuspended in suspension buffer (10 mM MgCl_2_, 10 mM MES, 50 μM acetosyringone) to OD_600_ = 0.8 and then co‐infiltrated into the leaves of *Nicotiana benthamiana* plants.

### ChIP‐qPCR

ChIP assays were performed using anti‐H3K27ac and anti‐H3K27me3 antibodies, with anti‐IgG antibody as a control. After ChIP‐qPCR analysis using the specific primers listed in Table S2, the degree of enrichment was calculated following normalization. The immunoprecipitation and qPCR analysis were performed three times.

### Yeast one‐hybrid assay

The full‐length coding sequence of *BpHST1* and the *BpCEL1* promoter (from 2000 bases upstream to 1000 bases downstream of the transcription start site) were cloned into the pGADT7 and pAbAi vectors, respectively. The *proBpCEL1*‐pAbAi construct was introduced into Y1H competent cells (WeiDi, Shanghai, China); positive yeast colonies were identified based on growth on synthetic defined medium lacking uracil (SD/–Ura) (TaKaRa, Beijing, China) containing 400 ng/mL aureobasidin A (AbA) at 30°C for approximately 3 d to determine the lowest AbA concentration that inhibited the growth of the yeast transformants. The pGADT7‐*BpHST1* and *proBpCEL1*‐pAbAi constructs were co‐introduced into Y1HGold yeast to test their interaction; pGADT7‐Rec‐53 and p53‐pABAi were used as the positive control, and pGADT7 and *proBpCEL1*‐pAbAi were used as the negative control. Yeast cultures were spotted on SD/−Leu medium with 400 ng/mL AbA and incubated at 30°C for 3 days.

### Statistical analysis

At least three independent samples were measured for each analysis, and the GraphPad Prism software was used for data analysis and visualization. Mean values for each measured parameter were compared using *t*‐test (*P < 0.05; **P < 0.01; ***P < 0.001).

## Author Contributions

ZZ and QZ conceived and designed the research. LH conducted laboratory experiments and edited the manuscript. BL revised the manuscript. YL provided experimental suggestions. All authors read and discussed the results and revised the manuscript.

## Conflict of Interest

The authors have not declared a conflict of interest.

## Supporting information


**Figure S1.** Sanger sequencing chromatogram of *BpHST1* gene editing. The DL‐1 sequence displays normal reference bases; the heterozygous mutation shows overlapping peaks (arrow) at the target site in the edited sequence; the homozygous mutation exhibits a deleted base (arrow); the protospacer adjacent motif (PAM) sequence; ‘NGG’ is highlighted in light red; the arrow positions represent the mutation sites; del. for deletion; ins for insertion. The ‘g^128 del 1^’ represent genomic 128 th deletion 1 bp, respectively. Leu: Leucine; Lys: Lysine; His: Histidine; Glu: Glutamic acid; Asp: Aspartic acid.
**Figure S2.** Analysis of subcellular localization of BpHST1. BpHST1 was localized to both the nucleus and cytoplasm. 35S: GFP was used as the negative control, fluorescent markers (GFP), DAPI staining (for nucleus), Bar = 50 μm.
**Figure S3.** Distribution and binding situation of histone peptide modification chips. (A) Appearance and lattice distribution of histone peptide modified chip. The red circles represent the specific binding sites of BpHST1, the left and right sides of the peptide array represent repetitions. (B) The top eight histone modification sites with the highest binding preference for BpHST1–GST. Candidate sites were ranked by normalized signal intensity, and the top eight high‑intensity spots are shown. The specificity factor represents the ratio of the normalized signal intensity of each target modification to the mean intensity of negative control spots (empty spots) on the same subarray.
**Figure S4.** Analysis of the H3K27ac and H3K27me3 ChIP‐seq. (A) Genome‐wide redistribution of H3K27ac and H3K27me3 histone marks. Vertical bars of the upper plot show the number of peaks, denoted by connected black circles below the histogram. (B) Scatter plot analysis of H3K27ac enrichment between DL‐1 and *bphst1*. (C) Scatter plot analysis of H3K27me3 enrichment between DL‐1 and *bphst1*.
**Figure S5.** IGV illustrating the reproducible peaks of H3K27ac and H3K27me3. Gray boxes, black boxes and black lines represent the 5′‐UTR, exons and introns, respectively. Transcription start sites (TSS) were presented with arrows, Bar = 200 bp.
**Figure S6.** Analysis of the RNA‐seq. (A) PCA of DL‐1, cr1 and cr2 leaves for the corresponding transcriptome data sets. The values were determined from log2‐transformed ion counts for the transcriptomics (*n* = 3 biologically independent samples) data sets. cr1: *bphst1‐5(−/−)*, cr2: *bphst1‐8(−/−)*. The circles outline biological replicates for each genotype. (B) Volcano plot showing the preferential accumulation of the differentially expressed genes of DL‐1 and cr1. The red scattered dots represent upregulated genes the blue scattered dots represent downregulated genes.
**Figure S7.** Analysis of H3K27ac CHIP‐seq. (A) Distribution of H3K27ac peaks in the birch genome. (B) Identification of enriched motifs in *BpHST1* binding peaks through MEME.
**Figure S8.** IGV illustrating the reproducible peaks of ChIP‐seq and RNA‐seq. (A–K) Representative IGV tracks for the cell‐wall‐related genes *BpTBL36*, *BpFLA11*, *BpFLA11‐3*, *BpBXL4*, *BpXTH32*, *BpPGX3*, *BpXTH16*, *BpCSLG3*, *BpPRX72*, *BpTBL19*, and *BpFLA11‐2*. All data were derived from 2‐month‐old wild‐type DL‐1 and *bphst1‐5* mutants, including H3K27ac and H3K27me3 ChIP‐seq profiles, RNA‐seq transcriptome data, and ChIP‐seq data from the *35S::BpHST1‐FLAG* overexpression lines. Gray boxes, black boxes and black lines represent the 5′‐UTR, exons and introns, respectively. Transcription start sites (TSS) were presented with arrows, Bar = 200 bp. From top to bottom, they are H3K27ac ChIP‐seq, H3K27me3 ChIP‐seq, RNA‐Seq, and *35S:: HST1::FLAG* ChIP‐seq. The light‐colored series represents the wild‐type DL‐1, the dark‐colored curves represent the *bphst1‐5* mutants, and the pink curve is the *35S:: HST1::FLAG* lines.
**Figure S9.** RT‐qPCR validation of candidate direct target genes of *BpHST1* in birch. (A) RT‐qPCR analysis of the expression of genes in cell‐wall organization or biogenesis. The data are shown as mean ± SD, *n* = 3. Asterisks represent significant differences (**P < 0.01, *t*‐test).
**Figure S10.** ChIP‐seq analysis of *35S:HST1‐FLAG*. (A) Heatmap showing the enrichment levels of Flag at ChIP‐identified regions and randomly selected genomic regions. (B) ChIP‐seq enrichment of *35S:HST1‐FLAG* at target genomic regions.
**Figure S11.** Schematic diagram of effector vectors and reporter vectors.
**Figure S12.** RT‐qPCR analysis of *BpCEL1* expression in 35S::*CEL1* transgenic lines. The date are means ± SD, *n* = 3. Asterisks represent significant differences (***P < 0.001, *t*‐test).
**Figure S13.** Contents of three major cell‐wall polymers. (A–C) The contents of lignin, cellulose and hemicellulose in DL‐1 and *35S:BpCEL1‐22* and *35S:BpCEL1‐25*. The data are shown as mean ± SD, *n* = 3. (*P < 0.05, ns: not significant; *t*‐test).
**Figure S14.** Contents of three major cell‐wall polymers. (A–C) The contents of lignin, cellulose and hemicellulose in DL‐1 and *bphst1‐5* and *bphst1‐8*. The data are shown as mean ± SD, *n* = 3. (*P < 0.05, **P < 0.01, ns: not significant; *t*‐test).
**Figure S15.** Longitudinal section at the third branch of DL‐1 and *BpCEL*1‐22# and *bphst1‐5*. (A, B) Lines with red denote the vertical lateral branch; Lines with black denote the vertical stem; β represents the branch angle of the paraffin section; Bar = 100 μm. (C) The branch angles of lateral branches in A. (D, E) The average length and width of DL‐1 and *BpCEL1‐22#* and *bphst1* lateral branch cells. Mean length and width of the within the two lines in A, respectively. (*P < 0.05, **P < 0.01, ***P < 0.001, ns: not significant, *t*‐test).

**Table S1.** MODified histone peptide array catalog.
**Table S2.** List of primers in this study.

## Data Availability

The data that support the findings of this study are available on request from the corresponding author. The data are not publicly available due to privacy or ethical restrictions.
