# Supplementary material for: The histone deacetylase BpHST1 influences branch angle in white birch (Betula platyphylla)
Source: Plant J. 2026 Sep 13;127(5):e71116. doi: 10.1111/tpj.71116 (PMC13571764; doi:10.1111/tpj.71116)
Supplement: Supplementary file 1 [file TPJ-127-0-s002.pdf]

Table S1. MODified Histone Peptide Array Catalog

MODified™ Histone Peptide Array Catalog						
**location**	**Peptide sequence**	**name**	**Mod1**	**Mod2**	**Mod 3**	**Mod 4**
A 1	A R T K Q T A R K S T G G K A P R K Q	H3 1-19	unmod			
A 2	A Rme2s T K Q T A R K S T G G K A P R K Q	H3 1-19	R2me2s			
A 3	A Rme2a T K Q T A R K S T G G K A P R K Q	H3 1-19	R2me2a			
A 4	A Cit T K Q T A R K S T G G K A P R K Q	H3 1-19	R2Citr			
A 5	A R pT K Q T A R K S T G G K A P R K Q	H3 1-19	T3P			
A 6	A R T Kme1 Q T A R K S T G G K A P R K Q	H3 1-19	K4me1			
A 7	A R T Kme2 Q T A R K S T G G K A P R K Q	H3 1-19	K4me2			
A 8	A R T Kme3 Q T A R K S T G G K A P R K Q	H3 1-19	K4me3			
A 9	A R T Kac Q T A R K S T G G K A P R K Q	H3 1-19	K4ac			
A10	A R T K Q T A Rme2s K S T G G K A P R K Q	H3 1-19	R8me2s			
A11	A R T K Q T A Rme2a K S T G G K A P R K Q	H3 1-19	R8me2a			
A12	A R T K Q T A Cit K S T G G K A P R K Q	H3 1-19	R8Citr			
A13	A R T K Q T A R Kme1 S T G G K A P R K Q	H3 1-19	K9me1			
A14	A R T K Q T A R Kme2 S T G G K A P R K Q	H3 1-19	K9me2			
A15	A R T K Q T A R Kme3 S T G G K A P R K Q	H3 1-19	K9me3			
A16	A R T K Q T A R Kac S T G G K A P R K Q	H3 1-19	K9ac			
A17	A R T K Q T A R K pS T G G K A P R K Q	H3 1-19	S10P			
A18	A R T K Q T A R K S pT G G K A P R K Q	H3 1-19	T11P			
A19	A R T K Q T A R K S T G G Kac A P R K Q	H3 1-19	K14ac			
A20	A Rme2s pT K Q T A R K S T G G K A P R K Q	H3 1-19	R2me2s	T3P		
A21	A Rme2s T Kme1 Q T A R K S T G G K A P R K Q	H3 1-19	R2me2s	K4me1		
A22	A Rme2s T Kme2 Q T A R K S T G G K A P R K Q	H3 1-19	R2me2s	K4me2		
A23	A Rme2s T Kme3 Q T A R K S T G G K A P R K Q	H3 1-19	R2me2s	K4me3		
A24	A Rme2s T Kac Q T A R K S T G G K A P R K Q	H3 1-19	R2me2s	K4ac		
B 1	A Rme2a pT K Q T A R K S T G G K A P R K Q	H3 1-19	R2me2a	T3P		
B 2	A Rme2a T Kme1 Q T A R K S T G G K A P R K Q	H3 1-19	R2me2a	K4me1		
B 3	A Rme2a T Kme2 Q T A R K S T G G K A P R K Q	H3 1-19	R2me2a	K4me2		
B 4	A Rme2a T Kme3 Q T A R K S T G G K A P R K Q	H3 1-19	R2me2a	K4me3		
B 5	A Rme2a T Kac Q T A R K S T G G K A P R K Q	H3 1-19	R2me2a	K4ac		
B 6	A Cit pT K Q T A R K S T G G K A P R K Q	H3 1-19	R2Citr	T3P		
B 7	A Cit T Kme1 Q T A R K S T G G K A P R K Q	H3 1-19	R2Citr	K4me1		
B 8	A Cit T Kme2 Q T A R K S T G G K A P R K Q	H3 1-19	R2Citr	K4me2		
B 9	A Cit T Kme3 Q T A R K S T G G K A P R K Q	H3 1-19	R2Citr	K4me3		
B10	A Cit T Kac Q T A R K S T G G K A P R K Q	H3 1-19	R2Citr	K4ac		
B11	A R pT Kme1 Q T A R K S T G G K A P R K Q	H3 1-19	T3P	K4me1		
B12	A R pT Kme2 Q T A R K S T G G K A P R K Q	H3 1-19	T3P	K4me2		
B13	A R pT Kme3 Q T A R K S T G G K A P R K Q	H3 1-19	T3P	K4me3		

**B14**	**A R pT Kac Q T A R K S T G G K A P R K Q**	**H3 1-19**	**T3P**	**K4ac**		
**B15**	**A Rme2s pT Kme1 Q T A R K S T G G K A P R K Q**	**H3 1-19**	**R2me2s**	**T3P**	**K4me1**	
**B16**	**A Rme2s pT Kme2 Q T A R K S T G G K A P R K Q**	**H3 1-19**	**R2me2s**	**T3P**	**K4me2**	
**B17**	**A Rme2s pT Kme3 Q T A R K S T G G K A P R K Q**	**H3 1-19**	**R2me2s**	**T3P**	**K4me3**	
**B18**	**A Rme2s pT Kac Q T A R K S T G G K A P R K Q**	**H3 1-19**	**R2me2s**	**T3P**	**K4ac**	
**B19**	**A Rme2a pT Kme1 Q T A R K S T G G K A P R K Q**	**H3 1-19**	**R2me2a**	**T3P**	**K4me1**	
**B20**	**A Rme2a pT Kme2 Q T A R K S T G G K A P R K Q**	**H3 1-19**	**R2me2a**	**T3P**	**K4me2**	
**B21**	**A Rme2a pT Kme3 Q T A R K S T G G K A P R K Q**	**H3 1-19**	**R2me2a**	**T3P**	**K4me3**	
**B22**	**A Rme2a pT Kac Q T A R K S T G G K A P R K Q**	**H3 1-19**	**R2me2a**	**T3P**	**K4ac**	
**B23**	**A R T K Q T A Rme2a Kme1 S T G G K A P R K Q**	**H3 1-19**	**R8me2s**	**K9me1**		
**B24**	**A R T K Q T A Rme2a Kme2 S T G G K A P R K Q**	**H3 1-19**	**R8me2s**	**K9me2**		
**C 1**	**A R T K Q T A Rme2a Kme3 S T G G K A P R K Q**	**H3 1-19**	**R8me2s**	**K9me3**		
**C 2**	**A R T K Q T A Rme2a Kac S T G G K A P R K Q**	**H3 1-19**	**R8me2s**	**K9ac**		
**C 3**	**A R T K Q T A Rme2a K pS T G G K A P R K Q**	**H3 1-19**	**R8me2s**	**S10P**		
**C 4**	**A R T K Q T A Rme2a K S pT G G K A P R K Q**	**H3 1-19**	**R8me2s**	**T11P**		
**C 5**	**A R T K Q T A Rme2a Kme1 S T G G K A P R K Q**	**H3 1-19**	**R8me2a**	**K9me1**		
**C 6**	**A R T K Q T A Rme2a Kme2 S T G G K A P R K Q**	**H3 1-19**	**R8me2a**	**K9me2**		
**C 7**	**A R T K Q T A Rme2a Kme3 S T G G K A P R K Q**	**H3 1-19**	**R8me2a**	**K9me3**		
**C 8**	**A R T K Q T A Rme2a Kac S T G G K A P R K Q**	**H3 1-19**	**R8me2a**	**K9ac**		
**C 9**	**A R T K Q T A Rme2a K pS T G G K A P R K Q**	**H3 1-19**	**R8me2a**	**S10P**		
**C10**	**A R T K Q T A Rme2a K S pT G G K A P R K Q**	**H3 1-19**	**R8me2a**	**T11P**		
**C11**	**A R T K Q T A Cit Kme1 S T G G K A P R K Q**	**H3 1-19**	**R8Citr**	**K9me1**		
**C12**	**A R T K Q T A Cit Kme2 S T G G K A P R K Q**	**H3 1-19**	**R8Citr**	**K9me2**		
**C13**	**A R T K Q T A Cit Kme3 S T G G K A P R K Q**	**H3 1-19**	**R8Citr**	**K9me3**		
**C14**	**A R T K Q T A Cit Kac S T G G K A P R K Q**	**H3 1-19**	**R8Citr**	**K9ac**		
**C15**	**A R T K Q T A Cit K pS T G G K A P R K Q**	**H3 1-19**	**R8Citr**	**S10P**		
**C16**	**A R T K Q T A Cit K S pT G G K A P R K Q**	**H3 1-19**	**R8Citr**	**T11P**		
**C17**	**A R T K Q T A R Kme1 pS T G G K A P R K Q**	**H3 1-19**	**K9me1**	**S10P**		
**C18**	**A R T K Q T A R Kme1 S pT G G K A P R K Q**	**H3 1-19**	**K9me1**	**T11P**		
**C19**	**A R T K Q T A R Kme1 S T G G Kac A P R K Q**	**H3 1-19**	**K9me1**	**K14ac**		
**C20**	**A R T K Q T A R Kme2 pS T G G K A P R K Q**	**H3 1-19**	**K9me2**	**S10P**		
**C21**	**A R T K Q T A R Kme2 S pT G G K A P R K Q**	**H3 1-19**	**K9me2**	**T11P**		
**C22**	**A R T K Q T A R Kme2 S T G G Kac A P R K Q**	**H3 1-19**	**K9me2**	**K14ac**		
**C23**	**A R T K Q T A R Kme3 pS T G G K A P R K Q**	**H3 1-19**	**K9me3**	**S10P**		
**C24**	**A R T K Q T A R Kme3 S pT G G K A P R K Q**	**H3 1-19**	**K9me3**	**T11P**		
**D 1**	**A R T K Q T A R Kme3 S T G G Kac A P R K Q**	**H3 1-19**	**K9me3**	**K14ac**		
**D 2**	**A R T K Q T A R Kac pS T G G K A P R K Q**	**H3 1-19**	**K9ac**	**S10P**		
**D 3**	**A R T K Q T A R Kac S pT G G K A P R K Q**	**H3 1-19**	**K9ac**	**T11P**		
**D 4**	**A R T K Q T A R Kac S T G G Kac A P R K Q**	**H3 1-19**	**K9ac**	**K14ac**		

D 5	A R T K Q T A R K pS pT G G K A P R K Q	H3 1-19	S10P	T11P		
D 6	A R T K Q T A R K pS T G G Kac A P R K Q	H3 1-19	S10P	K14ac		
D 7	A R T K Q T A R K S pT G G Kac A P R K Q	H3 1-19	T11P	K14ac		
D 8	A R T K Q T A Rme2s Kme1 pS T G G K A P R K Q	H3 1-19	R8me2s	K9me1	S10P	
D 9	A R T K Q T A Rme2s Kme2 pS T G G K A P R K Q	H3 1-19	R8me2s	K9me2	S10P	
D10	A R T K Q T A Rme2s Kme3 pS T G G K A P R K Q	H3 1-19	R8me2s	K9me3	S10P	
D11	A R T K Q T A Rme2s Kac pS T G G K A P R K Q	H3 1-19	R8me2s	K9ac	S10P	
D12	A R T K Q T A Rme2s Kme1 S pT G G K A P R K Q	H3 1-19	R8me2s	K9me1	T11P	
D13	A R T K Q T A Rme2s Kme2 S pT G G K A P R K Q	H3 1-19	R8me2s	K9me2	T11P	
D14	A R T K Q T A Rme2s Kme3 S pT G G K A P R K Q	H3 1-19	R8me2s	K9me3	T11P	
D15	A R T K Q T A Rme2s Kac S pT G G K A P R K Q	H3 1-19	R8me2s	K9ac	T11P	
D16	A R T K Q T A Rme2a Kme1 pS T G G K A P R K Q	H3 1-19	R8me2a	K9me1	S10P	
D17	A R T K Q T A Rme2a Kme2 pS T G G K A P R K Q	H3 1-19	R8me2a	K9me2	S10P	
D18	A R T K Q T A Rme2a Kme3 pS T G G K A P R K Q	H3 1-19	R8me2a	K9me3	S10P	
D19	A R T K Q T A Rme2a Kac pS T G G K A P R K Q	H3 1-19	R8me2a	K9ac	S10P	
D20	A R T K Q T A Rme2a Kme1 S pT G G K A P R K Q	H3 1-19	R8me2a	K9me1	T11P	
D21	A R T K Q T A Rme2a Kme2 S pT G G K A P R K Q	H3 1-19	R8me2a	K9me2	T11P	
D22	A R T K Q T A Rme2a Kme3 S pT G G K A P R K Q	H3 1-19	R8me2a	K9me3	T11P	
D23	A R T K Q T A Rme2a Kac S pT G G K A P R K Q	H3 1-19	R8me2a	K9ac	T11P	
D24	A R T K Q T A Rme2a Kme1 pS pT G G K A P R K Q	H3 1-19	R8me2a	K9me1	S10P	T11P
E 1	A R T K Q T A Rme2a Kme2 pS pT G G K A P R K Q	H3 1-19	R8me2a	K9me2	S10P	T11P
E 2	A R T K Q T A Rme2a Kme3 pS pT G G K A P R K Q	H3 1-19	R8me2a	K9me3	S10P	T11P
E 3	A R T K Q T A Rme2a Kac pS pT G G K A P R K Q	H3 1-19	R8me2a	K9ac	S10P	T11P
E 4	A Rme2s T Kme1 Q T A Rme2s K S T G G K A P R K Q	H3 1-19	R2me2s	K4me1	R8me2s	
E 5	A Rme2s T Kme2 Q T A Rme2s K S T G G K A P R K Q	H3 1-19	R2me2s	K4me2	R8me2s	
E 6	A Rme2s T Kme3 Q T A Rme2s K S T G G K A P R K Q	H3 1-19	R2me2s	K4me3	R8me2s	
E 7	A Rme2s T Kac Q T A Rme2s K S T G G K A P R K Q	H3 1-19	R2me2s	K4ac	R8me2s	
E 8	A Rme2a T Kme1 Q T A Rme2a K S T G G K A P R K Q	H3 1-19	R2me2a	K4me1	R8me2a	
E 9	A Rme2a T Kme2 Q T A Rme2a K S T G G K A P R K Q	H3 1-19	R2me2a	K4me2	R8me2a	
E10	A Rme2a T Kme3 Q T A Rme2a K S T G G K A P R K Q	H3 1-19	R2me2a	K4me3	R8me2a	
E11	A Rme2a T Kac Q T A Rme2a K S T G G K A P R K Q	H3 1-19	R2me2a	K4ac	R8me2a	
E12	A Rme2s T Kme1 Q T A R Kme1 S T G G K A P R K Q	H3 1-19	R2me2s	K4me1	K9me1	
E13	A Rme2s T Kme2 Q T A R Kme1 S T G G K A P R K Q	H3 1-19	R2me2s	K4me2	K9me1	
E14	A Rme2s T Kme3 Q T A R Kme1 S T G G K A P R K Q	H3 1-19	R2me2s	K4me3	K9me1	
E15	A Rme2s T Kac Q T A R Kme1 S T G G K A P R K Q	H3 1-19	R2me2s	K4ac	K9me1	
E16	A Rme2a T Kme1 Q T A R Kme2 S T G G K A P R K Q	H3 1-19	R2me2a	K4me1	K9me2	
E17	A Rme2a T Kme2 Q T A R Kme2 S T G G K A P R K Q	H3 1-19	R2me2a	K4me2	K9me2	
E18	A Rme2a T Kme3 Q T A R Kme2 S T G G K A P R K Q	H3 1-19	R2me2a	K4me3	K9me2	
E19	A Rme2a T Kac Q T A R Kme2 S T G G K A P R K Q	H3 1-19	R2me2a	K4ac	K9me2	

E20	A Rme2s T Kme1 Q T A R Kme3 S T G G K A P R K Q	H3 1-19	R2me2s	K4me1	K9me3	
E21	A Rme2s T Kme2 Q T A R Kme3 S T G G K A P R K Q	H3 1-19	R2me2s	K4me2	K9me3	
E22	A Rme2s T Kme3 Q T A R Kme3 S T G G K A P R K Q	H3 1-19	R2me2s	K4me3	K9me3	
E23	A Rme2s T Kac Q T A R Kme3 S T G G K A P R K Q	H3 1-19	R2me2s	K4ac	K9me3	
E24	A Rme2a T Kme1 Q T A R Kac S T G G K A P R K Q	H3 1-19	R2me2a	K4me1	K9ac	
F 1	A Rme2a T Kme2 Q T A R Kac S T G G K A P R K Q	H3 1-19	R2me2a	K4me2	K9ac	
F 2	A Rme2a T Kme3 Q T A R Kac S T G G K A P R K Q	H3 1-19	R2me2a	K4me3	K9ac	
F 3	A Rme2a T Kac Q T A R Kac S T G G K A P R K Q	H3 1-19	R2me2a	K4ac	K9ac	
F 4	A R T Kme1 Q T A Rme2s Kme1 S T G G K A P R K Q	H3 1-19	K4me1	R8me2s	K9me1	
F 5	A R T Kme2 Q T A Rme2s Kme1 S T G G K A P R K Q	H3 1-19	K4me2	R8me2s	K9me1	
F 6	A R T Kme3 Q T A Rme2s Kme1 S T G G K A P R K Q	H3 1-19	K4me3	R8me2s	K9me1	
F 7	A R T Kac Q T A Rme2s Kme1 S T G G K A P R K Q	H3 1-19	K4ac	R8me2s	K9me1	
F 8	A R T Kme1 Q T A Rme2a Kme1 S T G G K A P R K Q	H3 1-19	K4me1	R8me2a	K9me1	
F 9	A R T Kme2 Q T A Rme2a Kme1 S T G G K A P R K Q	H3 1-19	K4me2	R8me2a	K9me1	
F10	A R T Kme3 Q T A Rme2a Kme1 S T G G K A P R K Q	H3 1-19	K4me3	R8me2a	K9me1	
F11	A R T Kac Q T A Rme2a Kme1 S T G G K A P R K Q	H3 1-19	K4ac	R8me2a	K9me1	
F12	A R T Kme1 Q T A Rme2s Kme2 S T G G K A P R K Q	H3 1-19	K4me1	R8me2s	K9me2	
F13	A R T Kme2 Q T A Rme2s Kme2 S T G G K A P R K Q	H3 1-19	K4me2	R8me2s	K9me2	
F14	A R T Kme3 Q T A Rme2s Kme2 S T G G K A P R K Q	H3 1-19	K4me3	R8me2s	K9me2	
F15	A R T Kac Q T A Rme2s Kme2 S T G G K A P R K Q	H3 1-19	K4ac	R8me2s	K9me2	
F16	A R T Kme1 Q T A Rme2a Kme2 S T G G K A P R K Q	H3 1-19	K4me1	R8me2a	K9me2	
F17	A R T Kme2 Q T A Rme2a Kme2 S T G G K A P R K Q	H3 1-19	K4me2	R8me2a	K9me2	
F18	A R T Kme3 Q T A Rme2a Kme2 S T G G K A P R K Q	H3 1-19	K4me3	R8me2a	K9me2	
F19	A R T Kac Q T A Rme2a Kme2 S T G G K A P R K Q	H3 1-19	K4ac	R8me2a	K9me2	
F20	A R T Kme1 Q T A Rme2s Kme3 S T G G K A P R K Q	H3 1-19	K4me1	R8me2s	K9me3	
F21	A R T Kme2 Q T A Rme2s Kme3 S T G G K A P R K Q	H3 1-19	K4me2	R8me2s	K9me3	
F22	A R T Kme3 Q T A Rme2s Kme3 S T G G K A P R K Q	H3 1-19	K4me3	R8me2s	K9me3	
F23	A R T Kac Q T A Rme2s Kme3 S T G G K A P R K Q	H3 1-19	K4ac	R8me2s	K9me3	
F24	A R T Kme1 Q T A Rme2a Kme3 S T G G K A P R K Q	H3 1-19	K4me1	R8me2a	K9me3	
G 1	A R T Kme2 Q T A Rme2a Kme3 S T G G K A P R K Q	H3 1-19	K4me2	R8me2a	K9me3	
G 2	A R T Kme3 Q T A Rme2a Kme3 S T G G K A P R K Q	H3 1-19	K4me3	R8me2a	K9me3	
G 3	A R T Kac Q T A Rme2a Kme3 S T G G K A P R K Q	H3 1-19	K4ac	R8me2a	K9me3	
G 4	A R T Kme1 Q T A Rme2s Kac S T G G K A P R K Q	H3 1-19	K4me1	R8me2s	K9ac	
G 5	A R T Kme2 Q T A Rme2s Kac S T G G K A P R K Q	H3 1-19	K4me2	R8me2s	K9ac	
G 6	A R T Kme3 Q T A Rme2s Kac S T G G K A P R K Q	H3 1-19	K4me3	R8me2s	K9ac	
G 7	A R T Kac Q T A Rme2s Kac S T G G K A P R K Q	H3 1-19	K4ac	R8me2s	K9ac	
G 8	A R T Kme1 Q T A Rme2a Kac S T G G K A P R K Q	H3 1-19	K4me1	R8me2a	K9ac	
G 9	A R T Kme2 Q T A Rme2a Kac S T G G K A P R K Q	H3 1-19	K4me2	R8me2a	K9ac	
G10	A R T Kme3 Q T A Rme2a Kac S T G G K A P R K Q	H3 1-19	K4me3	R8me2a	K9ac	

G11	A R T Kac Q T A Rme2a Kac S T G G K A P R K Q	H3 1-19	K4ac	R8me2a	K9ac	
G12	A Rme2s T Kme1 Q T A Rme2s Kme1 S T G G K A P R K Q	H3 1-19	R2me2s	K4me1	R8me2s	K9me1
G13	A Rme2s T Kme2 Q T A Rme2s Kme1 S T G G K A P R K Q	H3 1-19	R2me2s	K4me2	R8me2s	K9me1
G14	A Rme2s T Kme3 Q T A Rme2s Kme1 S T G G K A P R K Q	H3 1-19	R2me2s	K4me3	R8me2s	K9me1
G15	A Rme2s T Kac Q T A Rme2s Kme1 S T G G K A P R K Q	H3 1-19	R2me2s	K4ac	R8me2s	K9me1
G16	A Rme2a T Kme1 Q T A Rme2s Kme1 S T G G K A P R K Q	H3 1-19	R2me2a	K4me1	R8me2s	K9me1
G17	A Rme2a T Kme2 Q T A Rme2s Kme1 S T G G K A P R K Q	H3 1-19	R2me2a	K4me2	R8me2s	K9me1
G18	A Rme2a T Kme3 Q T A Rme2s Kme1 S T G G K A P R K Q	H3 1-19	R2me2a	K4me3	R8me2s	K9me1
G19	A Rme2a T Kac Q T A Rme2s Kme1 S T G G K A P R K Q	H3 1-19	R2me2a	K4ac	R8me2s	K9me1
G20	A Rme2s T Kme1 Q T A Rme2s Kme2 S T G G K A P R K Q	H3 1-19	R2me2s	K4me1	R8me2s	K9me2
G21	A Rme2s T Kme2 Q T A Rme2s Kme2 S T G G K A P R K Q	H3 1-19	R2me2s	K4me2	R8me2s	K9me2
G22	A Rme2s T Kme3 Q T A Rme2s Kme2 S T G G K A P R K Q	H3 1-19	R2me2s	K4me3	R8me2s	K9me2
G23	A Rme2s T Kac Q T A Rme2s Kme2 S T G G K A P R K Q	H3 1-19	R2me2s	K4ac	R8me2s	K9me2
G24	A Rme2a T Kme1 Q T A Rme2s Kme2 S T G G K A P R K Q	H3 1-19	R2me2a	K4me1	R8me2s	K9me2
H 1	A Rme2a T Kme2 Q T A Rme2s Kme2 S T G G K A P R K Q	H3 1-19	R2me2a	K4me2	R8me2s	K9me2
H 2	A Rme2a T Kme3 Q T A Rme2s Kme2 S T G G K A P R K Q	H3 1-19	R2me2a	K4me3	R8me2s	K9me2
H 3	A Rme2a T Kac Q T A Rme2s Kme2 S T G G K A P R K Q	H3 1-19	R2me2a	K4ac	R8me2s	K9me2
H 4	A Rme2s T Kme1 Q T A Rme2s Kme3 S T G G K A P R K Q	H3 1-19	R2me2s	K4me1	R8me2s	K9me3
H 5	A Rme2s T Kme2 Q T A Rme2s Kme3 S T G G K A P R K Q	H3 1-19	R2me2s	K4me2	R8me2s	K9me3
H 6	A Rme2s T Kme3 Q T A Rme2s Kme3 S T G G K A P R K Q	H3 1-19	R2me2s	K4me3	R8me2s	K9me3
H 7	A Rme2s T Kac Q T A Rme2s Kme3 S T G G K A P R K Q	H3 1-19	R2me2s	K4ac	R8me2s	K9me3
H 8	A Rme2a T Kme1 Q T A Rme2s Kme3 S T G G K A P R K Q	H3 1-19	R2me2a	K4me1	R8me2s	K9me3
H 9	A Rme2a T Kme2 Q T A Rme2s Kme3 S T G G K A P R K Q	H3 1-19	R2me2a	K4me2	R8me2s	K9me3
H10	A Rme2a T Kme3 Q T A Rme2s Kme3 S T G G K A P R K Q	H3 1-19	R2me2a	K4me3	R8me2s	K9me3
H11	A Rme2a T Kac Q T A Rme2s Kme3 S T G G K A P R K Q	H3 1-19	R2me2a	K4ac	R8me2s	K9me3
H12	A Rme2s T Kme1 Q T A Rme2s Kac S T G G K A P R K Q	H3 1-19	R2me2s	K4me1	R8me2s	K9ac
H13	A Rme2s T Kme2 Q T A Rme2s Kac S T G G K A P R K Q	H3 1-19	R2me2s	K4me2	R8me2s	K9ac
H14	A Rme2s T Kme3 Q T A Rme2s Kac S T G G K A P R K Q	H3 1-19	R2me2s	K4me3	R8me2s	K9ac
H15	A Rme2s T Kac Q T A Rme2s Kac S T G G K A P R K Q	H3 1-19	R2me2s	K4ac	R8me2s	K9ac
H16	A Rme2a T Kme1 Q T A Rme2s Kac S T G G K A P R K Q	H3 1-19	R2me2a	K4me1	R8me2s	K9ac
H17	A Rme2a T Kme2 Q T A Rme2s Kac S T G G K A P R K Q	H3 1-19	R2me2a	K4me2	R8me2s	K9ac
H18	A Rme2a T Kme3 Q T A Rme2s Kac S T G G K A P R K Q	H3 1-19	R2me2a	K4me3	R8me2s	K9ac
H19	A Rme2a T Kac Q T A Rme2s Kac S T G G K A P R K Q	H3 1-19	R2me2a	K4ac	R8me2s	K9ac
H20	A Rme2s T Kme1 Q T A Rme2a Kme1 S T G G K A P R K Q	H3 1-19	R2me2s	K4me1	R8me2a	K9me1
H21	A Rme2s T Kme2 Q T A Rme2a Kme1 S T G G K A P R K Q	H3 1-19	R2me2s	K4me2	R8me2a	K9me1
H22	A Rme2s T Kme3 Q T A Rme2a Kme1 S T G G K A P R K Q	H3 1-19	R2me2s	K4me3	R8me2a	K9me1
H23	A Rme2s T Kac Q T A Rme2a Kme1 S T G G K A P R K Q	H3 1-19	R2me2s	K4ac	R8me2a	K9me1
H24	A Rme2a T Kme1 Q T A Rme2a Kme1 S T G G K A P R K Q	H3 1-19	R2me2a	K4me1	R8me2a	K9me1
I 1	A Rme2a T Kme2 Q T A Rme2a Kme1 S T G G K A P R K Q	H3 1-19	R2me2a	K4me2	R8me2a	K9me1

I 2	A Rme2a T Kme3 Q T A Rme2a Kme1 S T G G K A P R K Q	H3 1-19	R2me2a	K4me3	R8me2a	K9me1
I 3	A Rme2a T Kac Q T A Rme2a Kme1 S T G G K A P R K Q	H3 1-19	R2me2a	K4ac	R8me2a	K9me1
I 4	A Rme2s T Kme1 Q T A Rme2a Kme2 S T G G K A P R K Q	H3 1-19	R2me2s	K4me1	R8me2a	K9me2
I 5	A Rme2s T Kme2 Q T A Rme2a Kme2 S T G G K A P R K Q	H3 1-19	R2me2s	K4me2	R8me2a	K9me2
I 6	A Rme2s T Kme3 Q T A Rme2a Kme2 S T G G K A P R K Q	H3 1-19	R2me2s	K4me3	R8me2a	K9me2
I 7	A Rme2s T Kac Q T A Rme2a Kme2 S T G G K A P R K Q	H3 1-19	R2me2s	K4ac	R8me2a	K9me2
I 8	A Rme2a T Kme1 Q T A Rme2a Kme2 S T G G K A P R K Q	H3 1-19	R2me2a	K4me1	R8me2a	K9me2
I 9	A Rme2a T Kme2 Q T A Rme2a Kme2 S T G G K A P R K Q	H3 1-19	R2me2a	K4me2	R8me2a	K9me2
I10	A Rme2a T Kme3 Q T A Rme2a Kme2 S T G G K A P R K Q	H3 1-19	R2me2a	K4me3	R8me2a	K9me2
I11	A Rme2a T Kac Q T A Rme2a Kme2 S T G G K A P R K Q	H3 1-19	R2me2a	K4ac	R8me2a	K9me2
I12	A Rme2s T Kme1 Q T A Rme2a Kme3 S T G G K A P R K Q	H3 1-19	R2me2s	K4me1	R8me2a	K9me3
I13	A Rme2s T Kme2 Q T A Rme2a Kme3 S T G G K A P R K Q	H3 1-19	R2me2s	K4me2	R8me2a	K9me3
I14	A Rme2s T Kme3 Q T A Rme2a Kme3 S T G G K A P R K Q	H3 1-19	R2me2s	K4me3	R8me2a	K9me3
I15	A Rme2s T Kac Q T A Rme2a Kme3 S T G G K A P R K Q	H3 1-19	R2me2s	K4ac	R8me2a	K9me3
I16	A Rme2a T Kme1 Q T A Rme2a Kme3 S T G G K A P R K Q	H3 1-19	R2me2a	K4me1	R8me2a	K9me3
I17	A Rme2a T Kme2 Q T A Rme2a Kme3 S T G G K A P R K Q	H3 1-19	R2me2a	K4me2	R8me2a	K9me3
I18	A Rme2a T Kme3 Q T A Rme2a Kme3 S T G G K A P R K Q	H3 1-19	R2me2a	K4me3	R8me2a	K9me3
I19	A Rme2a T Kac Q T A Rme2a Kme3 S T G G K A P R K Q	H3 1-19	R2me2a	K4ac	R8me2a	K9me3
I20	A Rme2s T Kme1 Q T A Rme2a Kac S T G G K A P R K Q	H3 1-19	R2me2s	K4me1	R8me2a	K9ac
I21	A Rme2s T Kme2 Q T A Rme2a Kac S T G G K A P R K Q	H3 1-19	R2me2s	K4me2	R8me2a	K9ac
I22	A Rme2s T Kme3 Q T A Rme2a Kac S T G G K A P R K Q	H3 1-19	R2me2s	K4me3	R8me2a	K9ac
I23	A Rme2s T Kac Q T A Rme2a Kac S T G G K A P R K Q	H3 1-19	R2me2s	K4ac	R8me2a	K9ac
I24	A Rme2a T Kme1 Q T A Rme2a Kac S T G G K A P R K Q	H3 1-19	R2me2a	K4me1	R8me2a	K9ac
J 1	A Rme2a T Kme2 Q T A Rme2a Kac S T G G K A P R K Q	H3 1-19	R2me2a	K4me2	R8me2a	K9ac
J 2	A Rme2a T Kme3 Q T A Rme2a Kac S T G G K A P R K Q	H3 1-19	R2me2a	K4me3	R8me2a	K9ac
J 3	A Rme2a T Kac Q T A Rme2a Kac S T G G K A P R K Q	H3 1-19	R2me2a	K4ac	R8me2a	K9ac
J 4	A R K S T G G K A P R K Q L A T K A A R	H3 7-26	unmod			
J 5	A R K S T G G Kac A P R K Q L A T K A A R	H3 7-26	K14ac			
J 6	A R K pS T G G Kac A P R K Q L A T K A A R	H3 7-26	K14ac	S10P		
J 7	A R K S pT G G Kac A P R K Q L A T K A A R	H3 7-26	K14ac	T11P		
J 8	A R K S T G G K A P Rme2s K Q L A T K A A R	H3 7-26	R17me2s			
J 9	A R K S T G G K A P Rme2a K Q L A T K A A R	H3 7-26	R17me2a			
J10	A R K S T G G K A P Cit K Q L A T K A A R	H3 7-26	R17Citr			
J11	A R K S T G G K A P R Kac Q L A T K A A R	H3 7-26	K18ac			
J12	A R K S T G G Kac A P Rme2s K Q L A T K A A R	H3 7-26	K14ac	R17me2s		
J13	A R K S T G G Kac A P Rme2a K Q L A T K A A R	H3 7-26	K14ac	R17me2a		
J14	A R K S T G G Kac A P R Kac Q L A T K A A R	H3 7-26	K14ac	K18ac		
J15	A R K S T G G K A P Rme2s Kac Q L A T K A A R	H3 7-26	R17me2s	K18ac		
J16	A R K S T G G K A P Rme2a Kac Q L A T K A A R	H3 7-26	R17me2a	K18ac		

J17	A R K S T G G K A P Cit Kac Q L A T K A A R	H3 7-26	R17Citr	K18ac		
J18	A R K S T G G Kac A P Rme2s Kac Q L A T K A A R	H3 7-26	K14ac	R17me2s	K18ac	
J19	A R K S T G G Kac A P Rme2a Kac Q L A T K A A R	H3 7-26	K14ac	R17me2a	K18ac	
J20	P R K Q L A T K A A R K S A P A T G G	H3 16-35	unmod			
J21	P R K Q L A T K A A Rme2s K S A P A T G G	H3 16-35	R26me2s			
J22	P R K Q L A T K A A Rme2a K S A P A T G G	H3 16-35	R26me2a			
J23	P R K Q L A T K A A Cit K S A P A T G G	H3 16-35	R26Citr			
J24	P R K Q L A T K A A R Kme1 S A P A T G G	H3 16-35	K27me1			
K 1	P R K Q L A T K A A R Kme2 S A P A T G G	H3 16-35	K27me2			
K 2	P R K Q L A T K A A R Kme3 S A P A T G G	H3 16-35	K27me3			
K 3	P R K Q L A T K A A R Kac S A P A T G G	H3 16-35	K27ac			
K 4	P R K Q L A T K A A R K pS A P A T G G	H3 16-35	S28P			
K 5	P R K Q L A T K A A Rme2s Kme1 S A P A T G G	H3 16-35	R26me2s	K27me1		
K 6	P R K Q L A T K A A Rme2s Kme2 S A P A T G G	H3 16-35	R26me2s	K27me2		
K 7	P R K Q L A T K A A Rme2s Kme3 S A P A T G G	H3 16-35	R26me2s	K27me3		
K 8	P R K Q L A T K A A Rme2s Kac S A P A T G G	H3 16-35	R26me2s	K27ac		
K 9	P R K Q L A T K A A Rme2s K pS A P A T G G	H3 16-35	R26me2s	S28P		
K10	P R K Q L A T K A A Rme2a Kme1 S A P A T G G	H3 16-35	R26me2a	K27me1		
K11	P R K Q L A T K A A Rme2a Kme2 S A P A T G G	H3 16-35	R26me2a	K27me2		
K12	P R K Q L A T K A A Rme2a Kme3 S A P A T G G	H3 16-35	R26me2a	K27me3		
K13	P R K Q L A T K A A Rme2a Kac S A P A T G G	H3 16-35	R26me2a	K27ac		
K14	P R K Q L A T K A A Rme2a K pS A P A T G G	H3 16-35	R26me2a	S28P		
K15	P R K Q L A T K A A Cit Kme1 S A P A T G G	H3 16-35	R26Citr	K27me1		
K16	P R K Q L A T K A A Cit Kme2 S A P A T G G	H3 16-35	R26Citr	K27me2		
K17	P R K Q L A T K A A Cit Kme3 S A P A T G G	H3 16-35	R26Citr	K27me3		
K18	P R K Q L A T K A A Cit K pS A P A T G G	H3 16-35	R26Citr	S28P		
K19	P R K Q L A T K A A R Kme1 pS A P A T G G	H3 16-35	K27me1	S28P		
K20	P R K Q L A T K A A R Kme2 pS A P A T G G	H3 16-35	K27me2	S28P		
K21	P R K Q L A T K A A R Kme3 pS A P A T G G	H3 16-35	K27me3	S28P		
K22	P R K Q L A T K A A R Kac pS A P A T G G	H3 16-35	K27ac	S28P		
K23	P R K Q L A T K A A Rme2s Kme1 pS A P A T G G	H3 16-35	R26me2s	K27me1	S28P	
K24	P R K Q L A T K A A Rme2s Kme2 pS A P A T G G	H3 16-35	R26me2s	K27me2	S28P	
L 1	P R K Q L A T K A A Rme2s Kme3 pS A P A T G G	H3 16-35	R26me2s	K27me3	S28P	
L 2	P R K Q L A T K A A Rme2s Kac pS A P A T G G	H3 16-35	R26me2s	K27ac	S28P	
L 3	P R K Q L A T K A A Rme2a Kme1 pS A P A T G G	H3 16-35	R26me2a	K27me1	S28P	
L 4	P R K Q L A T K A A Rme2a Kme2 pS A P A T G G	H3 16-35	R26me2a	K27me2	S28P	
L 5	P R K Q L A T K A A Rme2a Kme3 pS A P A T G G	H3 16-35	R26me2a	K27me3	S28P	
L 6	P R K Q L A T K A A Rme2a Kac pS A P A T G G	H3 16-35	R26me2a	K27ac	S28P	
L 7	R K S A P A T G G V K K P H R Y R P G	H3 26-45	unmod			

L 8	R K S A P A T G G V Kme1 K P H R Y R P G	H3 26-45	K36me1			
L 9	R K S A P A T G G V Kme2 K P H R Y R P G	H3 26-45	K36me2			
L10	R K S A P A T G G V Kme3 K P H R Y R P G	H3 26-45	K36me3			
L11	R K S A P A T G G V Kac K P H R Y R P G	H3 26-45	K36ac			
L12	S G R G K G G K G L G K G G A K R H R	H4 1-19	unmod			
L13	pS G R G K G G K G L G K G G A K R H R	H4 1-19	S1P			
L14	S G Rme2s G K G G K G L G K G G A K R H R	H4 1-19	R3me2s			
L15	S G Rme2a G K G G K G L G K G G A K R H R	H4 1-19	R3me2a			
L16	S G R G Kac G G K G L G K G G A K R H R	H4 1-19	K5ac			
L17	S G R G K G G Kac G L G K G G A K R H R	H4 1-19	K8ac			
L18	S G R G K G G K G L G Kac G G A K R H R	H4 1-19	K12ac			
L19	S G R G K G G K G L G K G G A Kac R H R	H4 1-19	K16ac			
L20	pS G Rme2s G K G G K G L G K G G A K R H R	H4 1-19	S1P	R3me2s		
L21	pS G Rme2a G K G G K G L G K G G A K R H R	H4 1-19	S1P	R3me2a		
L22	pS G R G Kac G G K G L G K G G A K R H R	H4 1-19	S1P	K5ac		
L23	S G Rme2s G Kac G G K G L G K G G A K R H R	H4 1-19	R3me2s	K5ac		
L24	S G Rme2s G K G G Kac G L G K G G A K R H R	H4 1-19	R3me2s	K8ac		
M 1	S G Rme2a G Kac G G K G L G K G G A K R H R	H4 1-19	R3me2a	K5ac		
M 2	S G Rme2a G K G G Kac G L G K G G A K R H R	H4 1-19	R3me2a	K8ac		
M 3	S G R G Kac G G Kac G L G K G G A K R H R	H4 1-19	K5ac	K8ac		
M 4	S G R G K G G Kac G L G Kac G G A K R H R	H4 1-19	K8ac	K12ac		
M 5	S G R G K G G Kac G L G K G G A Kac R H R	H4 1-19	K8ac	K16ac		
M 6	S G R G K G G K G L G Kac G G A Kac R H R	H4 1-19	K12ac	K16ac		
M 7	pS G Rme2s G Kac G G K G L G K G G A K R H R	H4 1-19	S1P	R3me2s	K5ac	
M 8	pS G Rme2a G Kac G G K G L G K G G A K R H R	H4 1-19	S1P	R3me2a	K5ac	
M 9	S G Rme2s G Kac G G Kac G L G K G G A K R H R	H4 1-19	R3me2s	K5ac	K8ac	
M10	S G Rme2a G Kac G G Kac G L G K G G A K R H R	H4 1-19	R3me2a	K5ac	K8ac	
M11	S G R G Kac G G Kac G L G Kac G G A K R H R	H4 1-19	K5ac	K8ac	K12ac	
M12	S G R G K G G Kac G L G Kac G G A Kac R H R	H4 1-19	K8ac	K12ac	K16ac	
M13	pS G Rme2s G Kac G G Kac G L G K G G A K R H R	H4 1-19	S1P	R3me2s	K5ac	K8ac
M14	pS G Rme2a G Kac G G Kac G L G K G G A K R H R	H4 1-19	S1P	R3me2a	K5ac	K8ac
M15	S G Rme2s G Kac G G Kac G L G Kac G G A K R H R	H4 1-19	R3me2s	K5ac	K8ac	K12ac
M16	S G Rme2a G Kac G G Kac G L G Kac G G A K R H R	H4 1-19	R3me2a	K5ac	K8ac	K12ac
M17	S G R G Kac G G Kac G L G Kac G G A Kac R H R	H4 1-19	K5ac	K8ac	K12ac	K16ac
M18	G K G G A K R H R K V L R D N I Q G I T	H4 11-30	unmod			
M19	G Kac G G A K R H R K V L R D N I Q G I T	H4 11-30	K12ac			
M20	G K G G A Kac R H R K V L R D N I Q G I T	H4 11-30	K16ac			
M21	G K G G A K Rme2s H R K V L R D N I Q G I T	H4 11-30	R17me2s			
M22	G K G G A K Rme2a H R K V L R D N I Q G I T	H4 11-30	R17me2a			

M23	G K G G A K R H Rme2s K V L R D N I Q G I T	H4 11-30	R19me2s			
M24	G K G G A K R H Rme2a K V L R D N I Q G I T	H4 11-30	R19me2a			
N 1	G K G G A K R H R Kme1 V L R D N I Q G I T	H4 11-30	K20me1			
N 2	G K G G A K R H R Kme2 V L R D N I Q G I T	H4 11-30	K20me2			
N 3	G K G G A K R H R Kme3 V L R D N I Q G I T	H4 11-30	K20me3			
N 4	G K G G A K R H R Kac V L R D N I Q G I T	H4 11-30	K20ac			
N 5	G K G G A K R H R K V L Rme2a D N I Q G I T	H4 11-30	R24me2a			
N 6	G K G G A K R H R K V L Rme2s D N I Q G I T	H4 11-30	R24me2s			
N 7	G Kac G G A Kac R H R K V L R D N I Q G I T	H4 11-30	K12ac	K16ac		
N 8	G K G G A Kac Rme2s H R K V L R D N I Q G I T	H4 11-30	K16ac	R17me2s		
N 9	G K G G A Kac Rme2a H R K V L R D N I Q G I T	H4 11-30	K16ac	R17me2a		
N10	G K G G A Kac R H Rme2s K V L R D N I Q G I T	H4 11-30	K16ac	R19me2s		
N11	G K G G A Kac R H Rme2a K V L R D N I Q G I T	H4 11-30	K16ac	R19me2a		
N12	G K G G A Kac R H R Kme1 V L R D N I Q G I T	H4 11-30	K16ac	K20me1		
N13	G K G G A Kac R H R Kme2 V L R D N I Q G I T	H4 11-30	K16ac	K20me2		
N14	G K G G A Kac R H R Kme3 V L R D N I Q G I T	H4 11-30	K16ac	K20me3		
N15	G K G G A Kac R H R Kac V L R D N I Q G I T	H4 11-30	K16ac	K20ac		
N16	G Kac G G A Kac R H R Kme1 V L R D N I Q G I T	H4 11-30	K12ac	K16ac	K20me1	
N17	G Kac G G A Kac R H R Kme2 V L R D N I Q G I T	H4 11-30	K12ac	K16ac	K20me2	
N18	G Kac G G A Kac R H R Kme3 V L R D N I Q G I T	H4 11-30	K12ac	K16ac	K20me3	
N19	G Kac G G A Kac R H R Kac V L R D N I Q G I T	H4 11-30	K12ac	K16ac	K20ac	
N20	G K G G A K R H Rme2a Kme1 V L R D N I Q G I T	H4 11-30	R19me2a	K20me1		
N21	G K G G A K R H Rme2a Kme2 V L R D N I Q G I T	H4 11-30	R19me2a	K20me2		
N22	G K G G A K R H Rme2a Kme3 V L R D N I Q G I T	H4 11-30	R19me2a	K20me3		
N23	G K G G A K R H Rme2a Kac V L R D N I Q G I T	H4 11-30	R19me2a	K20ac		
N24	G K G G A K R H Rme2s Kme1 V L R D N I Q G I T	H4 11-30	R19me2s	K20me1		
O 1	G K G G A K R H Rme2s Kme2 V L R D N I Q G I T	H4 11-30	R19me2s	K20me2		
O 2	G K G G A K R H Rme2s Kme3 V L R D N I Q G I T	H4 11-30	R19me2s	K20me3		
O 3	G K G G A K R H Rme2s Kac V L R D N I Q G I T	H4 11-30	R19me2s	K20ac		
O 4	G K G G A K R H R Kme1 V L Rme2a D N I Q G I T	H4 11-30	R24me2a	K20me1		
O 5	G K G G A K R H R Kme2 V L Rme2a D N I Q G I T	H4 11-30	R24me2a	K20me2		
O 6	G K G G A K R H R Kme3 V L Rme2a D N I Q G I T	H4 11-30	R24me2a	K20me3		
O 7	G K G G A K R H R Kac V L Rme2a D N I Q G I T	H4 11-30	R24me2a	K20ac		
O 8	G K G G A K R H R Kme1 V L Rme2s D N I Q G I T	H4 11-30	R24me2s	K20me1		
O 9	G K G G A K R H R Kme2 V L Rme2s D N I Q G I T	H4 11-30	R24me2s	K20me2		
O10	G K G G A K R H R Kme3 V L Rme2s D N I Q G I T	H4 11-30	R24me2s	K20me3		
O11	G K G G A K R H R Kac V L Rme2s D N I Q G I T	H4 11-30	R24me2s	K20ac		
O12	S G R G K Q G G K A R A K A K S R S S	H2a 1-19	unmod			
O13	pS G R G K Q G G K A R A K A K S R S S	H2a 1-19	S1P			

O14	S G R G Kac Q G G K A R A K A K S R S S	H2a 1-19	K5ac			
O15	S G R G K Q G G Kac A R A K A K S R S S	H2a 1-19	K9ac			
O16	S G R G K Q G G K A R A Kac A K S R S S	H2a 1-19	K13ac			
O17	pS G R G Kac Q G G K A R A K A K S R S S	H2a 1-19	S1P	K5ac		
O18	pS G R G K Q G G Kac A R A K A K S R S S	H2a 1-19	S1P	K9ac		
O19	pS G R G K Q G G K A R A Kac A K S R S S	H2a 1-19	S1P	K13ac		
O20	S G R G Kac Q G G Kac A R A K A K S R S S	H2a 1-19	K5ac	K9ac		
O21	S G R G Kac Q G G K A R A Kac A K S R S S	H2a 1-19	K5ac	K13ac		
O22	S G R G K Q G G Kac A R A Kac A K S R S S	H2a 1-19	K9ac	K13ac		
O23	pS G R G Kac Q G G Kac A R A K A K S R S S	H2a 1-19	S1P	K5ac	K9ac	
O24	pS G R G Kac Q G G K A R A Kac A K S R S S	H2a 1-19	S1P	K5ac	K13ac	
P 1	pS G R G K Q G G Kac A R A Kac A K S R S S	H2a 1-19	S1P	K9ac	K13ac	
P 2	S G R G Kac Q G G Kac A R A Kac A K S R S S	H2a 1-19	K5ac	K9ac	K13ac	
P 3	pS G R G Kac Q G G Kac A R A Kac A K S R S S	H2a 1-19	S1P	K5ac	K9ac	K13ac
P 4	P D P A K S A P A P K K G S K K A V T	H2B 1-19	unmod			
P 5	P D P A Kac S A P A P K K G S K K A V T	H2B 1-19	K5ac			
P 6	P D P A K S A P A P K Kac G S K K A V T	H2B 1-19	K12ac			
P 7	P D P A K S A P A P K K G pS K K A V T	H2B 1-19	S14P			
P 8	P D P A K S A P A P K K G S Kac K A V T	H2B 1-19	K15ac			
P 9	P D P A Kac S A P A P K Kac G S K K A V T	H2B 1-19	K5ac	K12ac		
P10	P D P A Kac S A P A P K K G pS K K A V T	H2B 1-19	K5ac	S14P		
P11	P D P A Kac S A P A P K K G S Kac K A V T	H2B 1-19	K5ac	K15ac		
P12	P D P A K S A P A P K Kac G pS K K A V T	H2B 1-19	K12ac	S14P		
P13	P D P A K S A P A P K Kac G S Kac K A V T	H2B 1-19	K12ac	K15ac		
P14	P D P A K S A P A P K K G pS Kac K A V T	H2B 1-19	S14P	K15ac		
P15	P D P A Kac S A P A P K Kac G pS K K A V T	H2B 1-19	K5ac	K12ac	S14P	
P16	P D P A Kac S A P A P K Kac G S Kac K A V T	H2B 1-19	K5ac	K12ac	K15ac	
P17	P D P A Kac S A P A P K K G pS Kac K A V T	H2B 1-19	K5ac	S14P	K15ac	
P18	P D P A K S A P A P K Kac G pS Kac K A V T	H2B 1-19	K12ac	S14P	K15ac	
P19	P D P A Kac S A P A P K Kac G pS Kac K A V T	H2B 1-19	K5ac	K12ac	S14P	K15ac
P20	Bio A A N W S H P Q F E K A A	Biotin, control peptide				
P21	E Q K L I S E E D L A	c-myc tag				
P22	HAc	neg. contol				
P23	K Kme1 Kme2 Kme3 Kac R Rme2s R Rme2a R Cit K Kme1 Kac Kme3 R K	background 01				
P24	R Rme2s K Kme1 Kac R Rme2a Kme2 K Kme3 R Kme1 Rme2s K Kac R K	background 02				

Table S2. List of primers in this study

Primer	Primer sequence
BP-TUB-F	AACTCATCAGCGGCAAGGAA
BP-TUB-R	GTAGAAAGGACGCTGTTGTAGGG
M13R	CAGGAAACAGCTATGAC
PGX3-QRTF	CACTGGGAAAGGAACCATTGAAGG
PGX3-QRTR	CAGTACTCCTTCACAGCCATCG
TBL36-QRTF	GATTCTGCTCACTGGTGGACTCA
TBL36-QRTR	CTTCGAAGAGCAGTCATGGTGG
FLA11-QRTF	ACCAAATCAAAGGGCAGCTCGA
FLA11-QRTR	TTGACAAGGCCGGTCGAAATGT
XTH16-QRTF	TGTTGCTGGCAACTCTGCTGG
XTH16-QRTR	GGTTTCCAGATGATGGAGTATGTGTG
CSLG3-QRTF	ATCAGGGTAGAGAATGTTGTCGAG
CSLG3-QRTR	GTAGCTGACACTCGGAGCAGG
BXL4-QRTF	ACACAAATGGACTTAAGGTGGCTG
BXL4-QRTR	TTCCATTCGCCTCGGATGACT
XTH32-QRTF	GTGCTGCCATCAAACTCCACC
XTH32-QRTR	ATGAAACTTGACCTCCCTGCCA
TBL19-QRTF	TGCAATCCTTGATCTGCCTCCTC
TBL19-QRTR	AATACCGTCGGCCGGAAGAAC
FLA112-QRTF	TCCTCGAAAAGGCCCATGGGTA
FLA112-QRTR	GTCGGCACTGGATTGCTCAATG
FLA113-QRTF	CCAATCCAGCCCACCAATGC
FLA113-QRTR	AGTGCCCGGTTTAAGGTTCG
PRX72-QRTF	TCTCCCCTGCAAACCTCACCG
PRX72-QRTR	AATGTGGATCCCATCGGTATTGG
PGEX-WT-F	ATGAAGATCTTCAACTGGGTACATAGG
PGEX-WT-R	TCAGACTGTAGCACCTTGAGTTGG
PGEX-TS1-F	AATTCATGAAGATCTTCATCATACCGTCCTTAAGGATGGGTTAGC
PGEX-TS1-R	TCGAGCTAACCCATCCTTAAGGACGGTATGATGAAGATCTTCATG
PGEX-TS2-F	CCGCGTGGATCCCCGGAATTCATGAAGATCTTCAACTGGGTACATAGG
PGEX-TS2-R	GTCACGATGCGGCCGCTCGAGTCACTCCGCAGACCCCATAA
